# Ultrasensitive Detection of Porcine Epidemic Diarrhea Virus Infections Using Multivalent DNA Nanostructure‐Enabled Lateral Flow Assay

**DOI:** 10.1002/adhm.202504037

**Published:** 2026-06-23

**Authors:** Saurabh Umrao, Akhila Naru, Dhanush Gandavadi, Mengxi Zheng, Saraswathi Lanka, Hyeongjun Cho, Chau Nguyen Minh Hoang, Jianqiang Zhang, Ying Fang, Xing Wang

**Affiliations:** ^1^ Department of Bioengineering University of Illinois at Urbana‐Champaign Urbana Illinois USA; ^2^ Nick Holonyak Jr. Micro and Nanotechnology Laboratory University of Illinois at Urbana‐Champaign Urbana Illinois USA; ^3^ Carl R. Woese Institute for Genomic Biology University of Illinois at Urbana‐Champaign Urbana Illinois USA; ^4^ Department of Chemistry University of Illinois at Urbana‐Champaign Urbana Illinois USA; ^5^ Cancer Center at Illinois University of Illinois at Urbana‐Champaign Urbana Illinois USA; ^6^ Department of Pathobiology College of Veterinary Medicine University of Illinois at Urbana‐Champaign Urbana Illinois USA; ^7^ Veterinary Diagnostic Laboratory College of Veterinary Medicine University of Illinois at Urbana‐Champaign Urbana Illinois USA; ^8^ Department of Veterinary Diagnostic and Production Animal Medicine College of Veterinary Medicine Iowa State University Ames Iowa USA

**Keywords:** aptamer‐based diagnostics, DNA nanostructures, lateral flow assay (LFA), multivalent binding, porcine epidemic diarrhea virus (PEDV), pre‐clinical PEDV testing

## Abstract

Early, rapid on‐site detection of viral pathogens in livestock is critical to prevent disease spread, minimize economic losses, and safeguard food security. However, most current diagnostics are limited to centralized laboratories or require specialized equipment such as electrochemical readers, with a few hours to multi‐day turnaround times that hinder field deployment. Here, we introduce a DNA nanostructure‐enabled lateral flow assay (LFA) for rapid, ultrasensitive detection of porcine epidemic diarrhea virus (PEDV) directly on farms. This assay employs net‐shaped DNA nanostructures presenting PEDV‐specific aptamers in a multivalent configuration, yielding ∼1000‐fold greater binding avidity than free aptamers. The platform demonstrates the limit of detection (LOD) of ∼1.33 ng/mL for recombinant PEDV nucleocapsid protein and ∼278 viral copies per test in pig oral fluid samples, with results obtained within 10 min without requiring nucleic acid extraction or amplification. Validation with animal clinical samples (n = 102) from swine farms demonstrates an accuracy of 97.06%, sensitivity of 96.92%, and specificity of 97.30% (Ct ≤ 37.42), without cross‐reactivity against other swine viruses. This work presents a robust and field‐deployable diagnostic platform that combines the programmability of DNA nanotechnology with the operational simplicity of LFA. Beyond PEDV, the modular nature of the DNA‐Net platform is readily adaptable for detecting other veterinary and zoonotic pathogens, advancing disease surveillance, and One Health diagnostics.

## Graphical abstract

A multivalent DNA nanostructure‐enabled lateral flow assay was developed for rapid, ultrasensitive detection of porcine epidemic diarrhea virus (PEDV) nucleocapsid protein. Designer net‐shaped DNA nanostructures (DNA‐Net) presenting PEDV‐specific aptamers achieved ~1000‐fold enhanced binding, enabling detection of viral copies with Ct ≤ 37.42 within 10 min. The platform offers a simple, field‐deployable diagnostic tool for on‐farm PEDV surveillance.

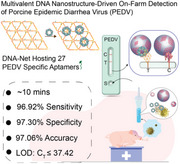

## 1 Introduction

Porcine epidemic diarrhea virus (PEDV) is an enteric Alphacoronavirus that causes acute, highly contagious diarrhea in swine, with particularly devastating effects on neonatal piglets, where infection often leads to 80%–100% mortality within affected litters [1]. Since its emergence in the 1970s, PEDV has spread across Europe, Asia, and North America, inflicting substantial economic losses on the pork industry. For instance, the introduction of PEDV into the United States in 2013 led to the loss of approximately 10% of the national herd within 18 months, resulting in economic damages exceeding USD 400 million [1, 2, 3]. Recent studies have further complicated PEDV epidemiology by demonstrating its potential to cross species barriers and infect rodents, including rats, which is concerning given that cross‐species coronavirus transmissions have triggered several major human and animal epidemics since 2003 [4]. Early and specific detection of PEDV is critical because its clinical signs are often indistinguishable from those caused by other enteric swine coronaviruses, such as transmissible gastroenteritis virus (TGEV) and porcine deltacoronavirus (PDCoV), necessitating laboratory confirmation for accurate differential diagnosis [5, 6].

Conventional diagnostic methods for PEDV include virus isolation, immunofluorescence assays, enzyme‐linked immunosorbent assays [6, 7], and nucleic acid amplification‐based tests such as reverse transcription‐polymerase chain reaction (RT‐PCR) and quantitative real‐time RT‐PCR (qRT‐PCR) [8, 9, 10]. Among these, RT‐PCR and qRT‐PCR are considered gold‐standard due to their high analytical sensitivity and specificity, often detecting as low as 10 viral RNA copies per reaction. However, these methods require centralized laboratory infrastructure, skilled personnel, and thermal cycling instrumentation, resulting in sample‐to‐result turnaround times of 2–4 days when factoring in sample transport and batching. To overcome the dependence on thermal cycling, isothermal amplification methods such as reverse transcription loop‐mediated isothermal amplification (RT‐LAMP) and recombinase polymerase amplification (RT‐RPA) have been developed, enabling rapid amplification at constant temperature [11, 12, 13, 14, 15]. For example, RT‐LAMP assays targeting PEDV nucleic acids have achieved sensitivities comparable to RT‐PCR within 40–60 min [13]. Despite their promise, these assays still require nucleic acid extraction and specialized reagents, limiting their practicality for on‐site deployment in farm environments with minimal resources.

Lateral flow immunoassays (LFIAs) address many of these limitations by offering significant advantages for point‐of‐care diagnostics. Their low cost, operational simplicity, rapid readouts within minutes, and independence from electricity or complex instrumentation make them particularly attractive for pen‐side use [16, 17, 18, 19, 20, 21]. Recent PEDV antigen LFIAs employing colloidal gold nanoparticles or latex bead reporters have demonstrated detection sensitivities ranging from 10^4^ to 10^5^ TCID_50_/mL, with clinical agreement rates of approximately 74%–96% compared to RT‐PCR [21, 22, 23, 24]. However, antibody‐based LFIAs face intrinsic limitations: (i) antibodies are prone to thermal degradation, necessitating cold chain logistics; (ii) batch‐to‐batch variability during production affects assay reproducibility; and (iii) relatively low affinity can restrict assay sensitivity, leading to false negatives particularly in samples with low viral loads during early infection or environmental surveillance [25, 26].

Recent advances have highlighted aptamer‐based diagnostics as a promising alternative to overcome the limitations of antibody‐dependent assays [27]. Aptamers are short, single‐stranded DNA or RNA oligonucleotides selected through systematic evolution of ligands by exponential enrichment (SELEX) to bind viral antigens (such as the PEDV spike or nucleocapsid proteins) with high affinity and specificity [28]. Compared to antibodies, aptamers offer multiple advantages such as chemical stability, low‐cost synthesis, and easy modification with labels or nanostructures [29, 30]. Notably, Li et al. [31]. reported the first PEDV‐specific DNA aptamer targeting the nucleocapsid (N) protein [32, 33, 34], a highly conserved and abundantly expressed viral structural protein involved in RNA binding and packaging. The group developed an electrochemical aptasensor integrating this aptamer, achieving a limit of detection of ∼0.37 µg/mL within 1 h, with 83% diagnostic sensitivity and 100% specificity compared to RT‐PCR in swine oral fluid samples. This work demonstrated the feasibility of aptamer‐based rapid diagnostics for PEDV without requiring nucleic acid extraction or amplification, paving the way for pen‐side deployment. However, achieving the ultrahigh sensitivity required to detect low viral loads during early infection still remains a major challenge for both lateral flow and aptamer‐based assays.

**SCHEME 1:**
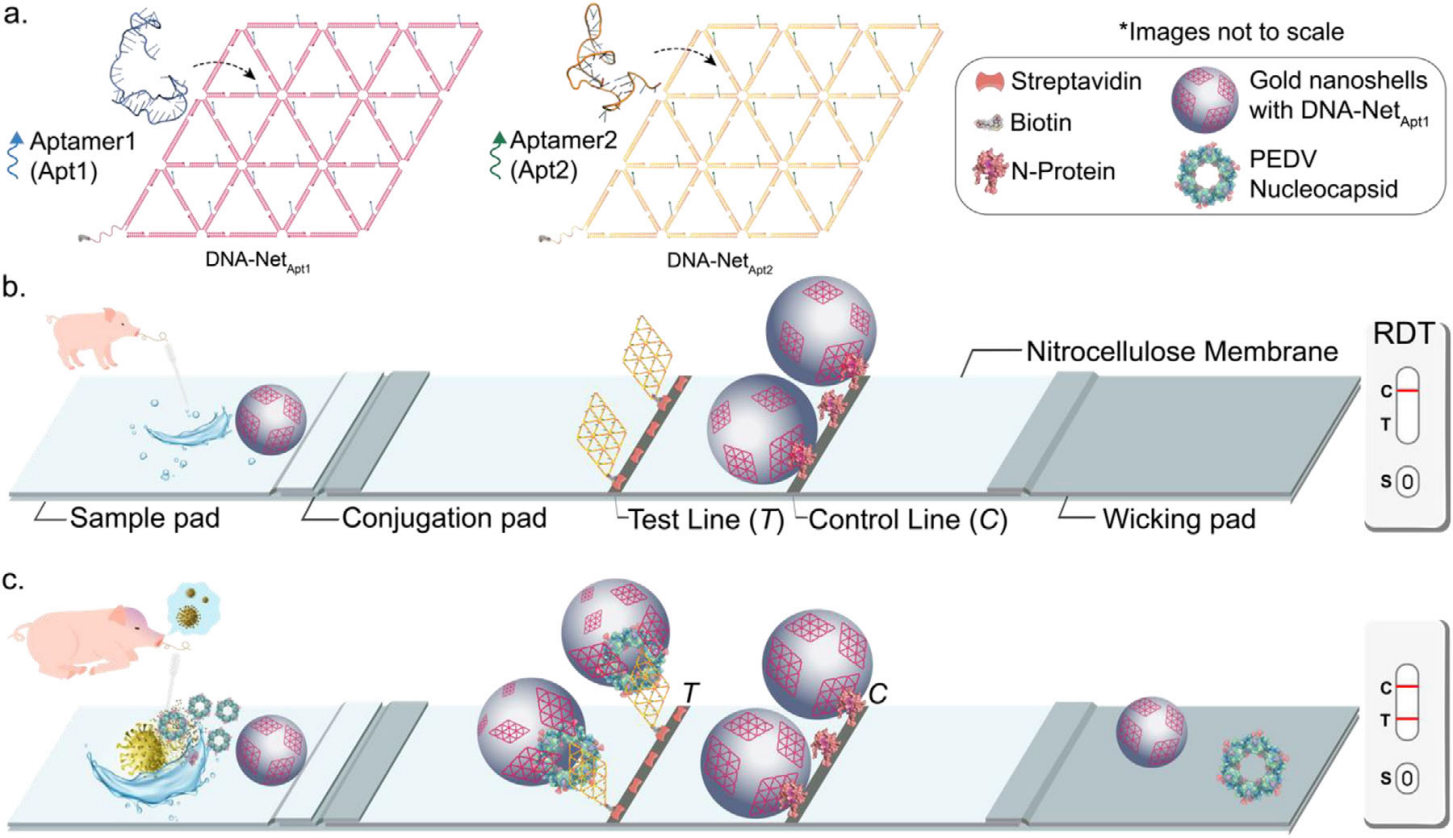
Schematic of the lateral flow assay (LFA) device using DNA‐Net constructs for PEDV‐N protein detection. (a) Aptamers 1 and 2 (referred as Apt1 and Apt2) were separately incorporated into trimeric clusters on DNA‐Net scaffolds to generate two distinct constructs: DNA‐Net_Apt1_ and DNA‐Net_Apt2_. The secondary structures of the DNA aptamers were predicted using the 3dDNA webserver [40]. (b) The DNA‐Net_Apt1_ construct was labeled with gold nanoshells (AuNS) and used as a reporter or detector probe (AuNS‐Net_Apt1_), while the DNA‐Net_Apt2_ construct (capture probe) was immobilized on the test line (T). Recombinant PEDV‐N protein was anchored on the control line to validate assay performance. (c) In the presence of the virus, lysis of the viral particles releases the de‐enveloped N protein, which forms a sandwich complex with both the reporter probe (AuNS‐Net_Apt1_) and the capture probe (DNA‐Net_Apt2_) on the test line. This results in a visible gray colored signal at the test line, which can be observed by the naked eye or quantified using a portable colorimetric reader. Regardless of the presence or absence of viral particles, the AuNS‐Net_Apt1_ reporter probe binds to the N protein on the control line, producing a high‐contrast gray colored signal to confirm assay functionality.

To address this challenge, innovative strategies leveraging DNA nanotechnology have emerged. For example, designer DNA nanostructures such as DNA Star [35, 36], DNA NanoGripper [37], and DNA‐Net [38] can host multiple molecular recognition elements (e.g., aptamers) arranged with nanoscale spatial precision. This multivalency effect enhances target binding through cooperative interactions, dramatically increasing effective affinity, and enabling multivalent recognition of complex targets like viruses. For instance, a net‐shaped DNA nanostructure (DNA‐Net) presenting trimeric clusters of aptamers targeting the SARS‐CoV‐2 spike protein achieved detection limits of 1000 viral copies of SARS‐CoV‐2 virus in lateral flow formats [39]. While such multivalency concept has been explored previously, this work extends it to a distinct mechanistic and translational setting by targeting a conserved antigen and implementing a dual aptamer sandwich assay for rapid diagnostics of PEDV (Table S1). Briefly, in this study, we leveraged aptamer‐integrated multivalent DNA nanostructures to develop an ultrasensitive, rapid, and field‐deployable diagnostic platform for PEDV detection. Using computational docking and structural modeling, we learned that two candidate aptamers (Apt1 and Apt2) bind to distinct domains of the PEDV nucleocapsid (N) protein. We used this information to devise a sandwich‐based detection strategy employing two sets of DNA‐Net constructs (DNA‐Net_Apt1_ and DNA‐Net_Apt2_), each functionalized with two distinct aptamers targeting separate epitopes on the PEDV‐N protein. We then used surface plasmon resonance (SPR) technique to compare the binding affinity of free aptamers to that of aptamers displayed on DNA‐Net constructs. Remarkably, the DNA‐Net constructs exhibited roughly a 1000‐fold improvement in apparent binding avidity over the free aptamers, underscoring the strong avidity conferred by multivalent interactions. We next incorporated these DNA‐Net constructs into a lateral flow assay (LFA) device. One DNA‐Net_Apt1_ construct was conjugated to gold nanoshell particles to serve as a reporter probe, while another DNA‐Net_Apt2_ construct was immobilized on the test strip as the capture agent (Scheme 1). This multivalent aptamer sandwich format enabled a PEDV‐N protein detection limit of 1.33 ng/mL in pig oral fluid, which is approximately 300‐fold more sensitive than previously reported lateral flow assays (∼0.37 µg/mL in saliva‐based conditions) [31] and ∼41‐fold more sensitive than a commercial PEDV antigen LFA (Limit of Detection of ∼0.055 µg/mL) [23]. We further demonstrated that the device could detect live virus, with a limit of detection of about 278 viral copies per test, which is a clinically relevant sensitivity threshold. Finally, we validated the platform using swine farm samples (*n* = 102) with different farm sources. The LFA device achieved a diagnostic accuracy of 97.06%, sensitivity of 96.92%, specificity of 97.30% in this cohort. Importantly, the assay delivers results within 10 min without requiring complex chemistry or expensive hardware. This work represents a significant advancement toward robust, scalable, and affordable diagnostic tools for PEDV surveillance and outbreak management, ultimately contributing to more resilient and responsive livestock health systems.

## 2 Results and Discussion

We employed two previously reported [31] aptamers (PEA1‐3, referred to as Apt1, and PEA2, referred to as Apt2) in combination with net‐shaped designer DNA nanostructure (DNA‐Net) constructs for the specific and sensitive detection of PEDV‐N protein. The sequences of these aptamers are provided in the Materials and Methods Section of the Supporting Information.

### 2.1 Molecular Docking Reveals Distinct Binding Sites of PEDV Aptamers

**FIGURE 1:**
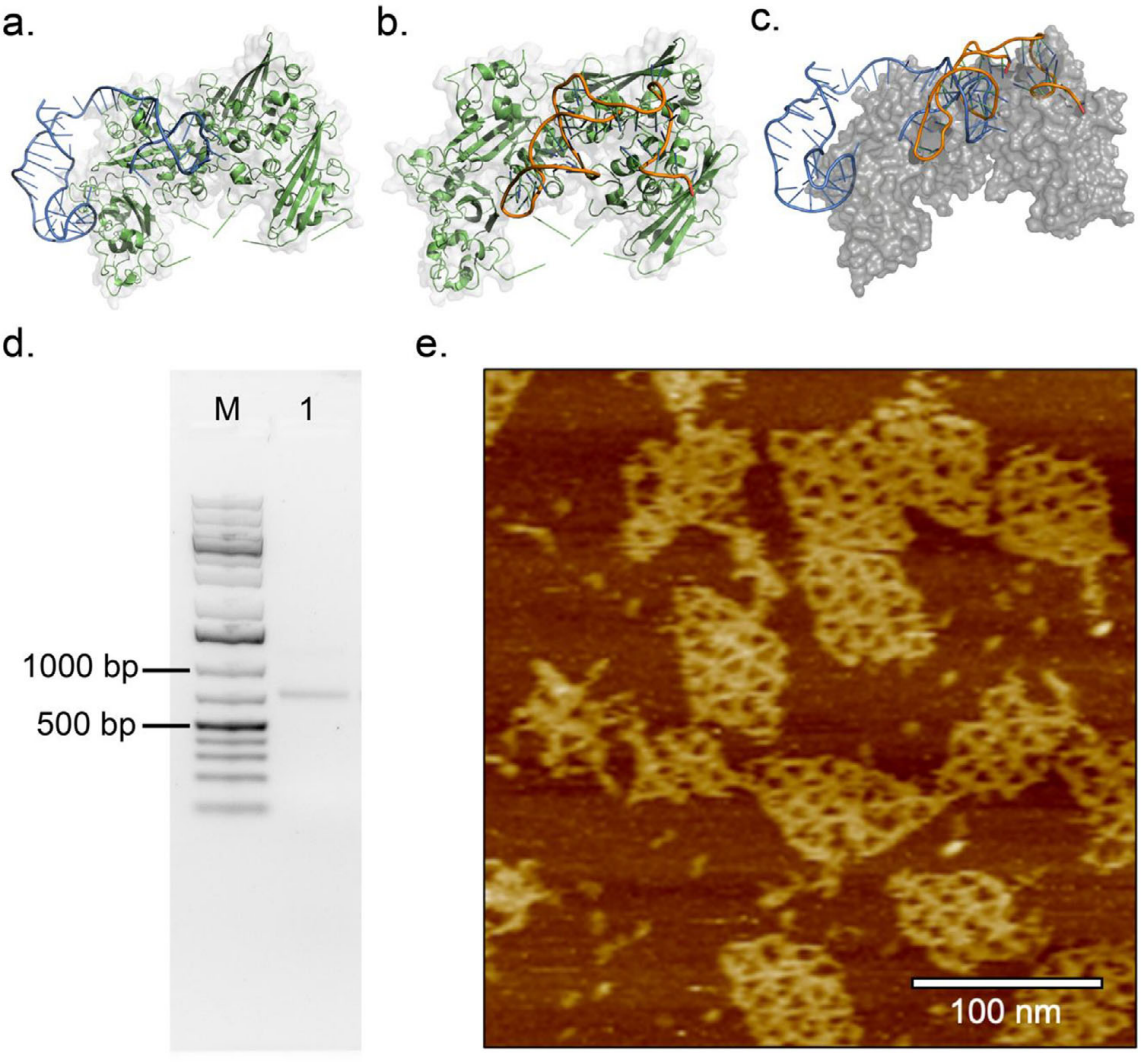
Molecular docking of DNA aptamers with PEDV‐N protein, and synthesis and characterization of DNA‐Net constructs. (a) Docking model showing Apt1 (marine color) bound to a specific site on the N protein (light green color), highlighting its stem‐loop‐like binding orientation. (b) Docking model of Apt2 (orange color) interacting with a distinct site on the N protein, consistent with its G‐quadruplex‐like folding conformation. (c) Superimposed structures of the Apt1–N protein and Apt2–N protein complexes, illustrating that the two aptamers engage non‐overlapping binding regions on the protein surface (shown in light gray color). Docking simulations were performed using HADDOCK and visualized in PyMOL. (d) The successful assembly of the DNA Net was verified using 1% agarose gel electrophoresis (AGE). (e) An atomic force microscopy (AFM) image further visualizes the DNA‐Net structure. Scale bar: 100 nm.

To inform the assay design strategy, we first performed molecular docking studies to investigate the binding footprints and interaction modes of both aptamers with the PEDV‐N protein. Using open access tools such as 3dDNA webserver [40, 41], we first generated the energy‐minimized three‐dimensional structures of the DNA aptamers which revealed that Apt1 adopts a stable stem‐loop‐like configuration (Figure S1a), whereas Apt2 exhibits a potential G‐quadruplex folded structure (Figure S1b). These observed structural differences suggested the possibility of distinct binding modes to the N protein. To explore this, we performed molecular docking of both aptamers with the PEDV‐N protein using the High Ambiguity Driven biomolecular DOCKing (HADDOCK) platform [42]. The resulting complexes were further analyzed using the Protein Ligand Interaction Profiler (PLIP) webserver [43] to identify key molecular interactions. As summarized in Tables S2 and S3, both aptamers engage the N protein through a combination of hydrogen bonding, salt bridges, and hydrophobic interactions (Figure 1a,b). Notably, the analysis revealed that Apt1 and Apt2 bind to distinct, non‐overlapping regions on the N protein surface, likely driven by their differing secondary and tertiary structures (Figure 1c). These insights supported the rationale for employing both aptamers in subsequent assay development.

### 2.2 Design Rationale, Assembly, and Characterization of Multivalent DNA‐Net Constructs

To leverage the benefits of multivalency for enhanced target binding, we engineered a 45 nm × 45 nm DNA‐Net platform utilizing a programmable multilayer Designer DNA Nanostructure (DDN) approach, which allows precise nanoscale spatial organization of trimeric molecular binders such as N protein‐specific aptamers. PEDV nucleocapsid protein is a multimeric RNA‐binding protein, which contains folded domains connected by intrinsically disordered, flexible regions and undergoes RNA‐dependent self‐association into heterogeneous conformations and oligomerization states [44, 45, 46]. To choose an aptamer spacing that is structurally reasonable yet compatible with this conformational variability, we used the PEDV N protein C‐terminal dimerization (CTD) domain as a physical length scale. The PEDV N CTD forms a stable homodimer (PDB 8WQK) spanning approximately 4.8 nm in maximal dimension, which provides a practical lower bound for multivalent engagement without steric interference. Accordingly, we selected an inter‐aptamer spacing of 6 nm, modestly larger than the CTD dimer span, to reduce steric crowding when two aptamers bind adjacent N surfaces while still maintaining proximity sufficient for avidity rather than independent monovalent interactions. We implemented this spacing in a 2D triangular trimer configuration on the DNA‐Net scaffold to increase local effective concentration and provide multiple ligand orientations, thereby improving capture robustness for a target whose accessible epitopes can shift with RNA binding and oligomerization. This design is further consistent with reported coronavirus N RNA assemblies forming vRNP‐like units on the order of 10 to 15 nm, which defines an upper bound local neighborhood scale over which multivalent co‐engagement is feasible [47]. Taken together, these constraints motivate a few‐nm to ∼10 nm design window and place our 6 nm spacing in a physically and biologically reasonable regime that balances steric accessibility with multivalent co‐engagement. Consequently, the DNA‐Net was designed to display up to 27 aptamers per construct, generating two configurations: DNA‐Net_Apt1_ and DNA‐Net_Apt2_ (Scheme 1). The assembly of the DNA‐Net constructs was verified via 1% agarose gel electrophoresis (AGE), which demonstrated clear and dominant bands in lane 1 corresponding to the successfully assembled nanostructures, indicative of high assembly efficiency and structural integrity (Figure 1d). Furthermore, atomic force microscopy (AFM) imaging was employed to assess the geometry and morphological fidelity of the constructs. Due to their thin, tile‐like architecture, DNA‐Net constructs are susceptible to mechanical deformation or structural disruption during deposition onto mica surfaces for AFM sample preparation. Despite this challenge, the AFM results revealed well‐defined, lattice‐like DNA‐Net nanostructures with dimensions consistent with the design specifications, confirming successful synthesis and folding (Figure 1e).

### 2.3 Surface Plasmon Resonance (SPR) Reveals Enhanced Binding Affinity of Multivalent DNA‐Net Aptamer Constructs compared to Free aptamers

**FIGURE 2:**
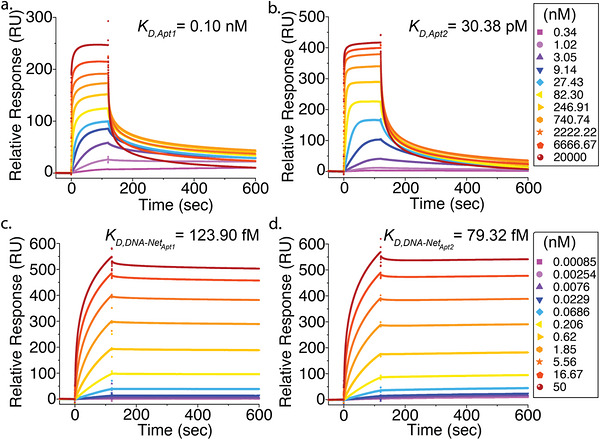
Surface plasmon resonance (SPR) analysis of binding affinities of monovalent aptamers and aptamers functionalized DNA‐Net constructs to PEDV‐N protein. Increasing concentrations of free aptamers (0.34 nm–20 µm) were injected over recombinant PEDV‐N protein immobilized on a CM5 sensor surface to determine dissociation constants (*K_D_*) for (a) Aptamer 1 and (b) Aptamer 2. For multivalent constructs, increasing concentrations of (c) DNA‐Net_Apt1_ and (d) DNA‐Net_Apt2_ (0.00085 to 50 nm) were injected over the same N protein surface. The resulting sensorgrams were used to determine binding kinetic parameters, including the association rate constant (ka), dissociation rate constant (kd), and equilibrium dissociation constant (*K_D_* = kd/ka), as summarized in Table S4. Parameters were obtained by locally fitting the full association and dissociation phases using a 1:1 Langmuir binding model in BIAevaluation software. Experiments were repeated using *n* = 3 biologically independent samples with similar results.

We employed SPR assays to evaluate the binding affinities of monomeric aptamers (Apt1 and Apt2), and their multivalent counterparts anchored on DNA‐Net constructs (DNA‐Net_Apt1_ and DNA‐Net_Apt2_) toward the PEDV‐N protein. For these experiments, 50 µg/mL of the recombinant PEDV‐N protein was immobilized on the flow cell of the SPR sensor chip and increasing concentrations of each analyte were injected. Concentration ranges for the monomeric aptamers were 0.34 nM to 20 µM, while DNA‐Net_Apt1_ and DNA‐Net_Apt2_ constructs were tested in the range of 0.00085–50 nM. Both free aptamers exhibited strong binding to the N protein, with dissociation constants (*K_D_*) of 0.10 nM for Apt1 and 30.38 pM for Apt2 (Figure 2a,b). Remarkably, the DNA‐Net constructs demonstrated approximately 1000‐fold enhancement in binding affinity compared to their monomeric counterparts, with *K_D_* values of 123.90 fM for DNA‐Net_Apt1_ and 79.32 fM for DNA‐Net_Apt2_ (Figure 2c,d). This dramatic improvement suggests the improved multivalent effect achieved through aptamer arrangement on the DNA‐Net scaffold.

To confirm that the observed strong binding affinities were specific to the aptamer–N protein interactions, several controls were included. As a positive control, a monoclonal antibody (mAb) against the N protein was tested, exhibiting a *K_D_* of 60.32 pM (Figure S2a). Negative controls included a concentration gradient of a non‐target influenza‐specific aptamer [48] (Figure S2b), unmodified DNA‐Net (Figure S2c), and DNA‐Net functionalized with the influenza‐specific aptamer using the same methodology employed for the DNA‐Net_Apt_ constructs (Figure S2d). None of these negative controls exhibited measurable binding to the PEDV‐N protein, confirming the specificity of the DNA‐Net_Apt1and2_ constructs. Overall, these results demonstrate that DNA‐Net‐mediated multivalent display of aptamers confers a substantial increase in binding affinity toward the PEDV‐N protein, highlighting the potential of this platform for ultrasensitive viral detection applications.

Next, to experimentally validate the sandwich binding mode (for downstream experiment design), we complemented molecular docking with sequential injection‐based SPR assays. In this format, Apt2 was first immobilized on the sensor surface, followed by serial injection of recombinant PEDV‐N protein and then Apt1. Injection of N protein produced a clear increase in resonance units, confirming its capture by surface bound Apt2, and after a brief buffer wash to remove unbound protein, subsequent Apt1 injection yielded an additional pronounced response increase (Figure S3a). This second binding step is expected only if Apt1 can bind the Apt2 captured N protein complex, providing direct evidence for non‐overlapping epitope recognition and formation of an Apt2–N protein–Apt1 sandwich complex. As a negative control, a non‐target influenza aptamer injected in place of Apt1 produced minimal additional response, confirming that the secondary signal is sequence specific and not driven by nonspecific adsorption.

Because our assay relies on integrating individual aptamers in a multivalent DNA‐Net context, we further verified that the sandwich configuration is maintained after DNA‐Net integration. We therefore performed an analogous sequential injection SPR experiment in which DNA‐Net_Apt2_ was immobilized on the sensor surface, followed by serial injection of PEDV‐N protein and then DNA‐Net_Apt1_. N protein again generated a robust binding response on the DNA‐Net_Apt2_ surface, and injection of DNA‐Net_Apt1_ produced an additional increase in resonance units, demonstrating that DNA Net presentation preserves epitope accessibility and supports the DNA‐Net_Apt2_–N protein– DNA‐Net_Apt1_ sandwich configuration (Figure S3b). A DNA‐Net control bearing non‐target influenza aptamers showed minimal response throughout the injection sequence, indicating that the observed binding is not due to nonspecific interactions with the DNA Net scaffold. Collectively, these SPR experiments provide direct mechanistic validation that both the free aptamers and the DNA Net displayed aptamers retain the required sandwich binding mode, strengthening the basis of the LFA detection mechanism beyond molecular docking predictions.

Of note, the reported 3‐log‐fold avidity enhancement was evaluated using SPR as the primary biophysical technique. SPR is a well‐established, quantitative, and widely used method for characterizing biomolecular interactions, including multivalent binding and avidity‐driven effects, and was particularly well suited here for comparative analysis of monovalent versus multivalent binding kinetics [49]. Nevertheless, additional affinity measurement techniques could further strengthen mechanistic interpretation, especially with respect to the structural and single‐event basis of the observed enhancement. Future studies using such complementary approaches may provide deeper insight into the molecular determinants of multivalent binding on the DNA‐Net scaffold.

### 2.4 Design and Characterization of DNA‐Net Aptamer‐based LFA Capture and Reporter Components

For the development of LFA‐based rapid diagnostics, it is essential to integrate two key components: a capture probe immobilized on the test line to bind with the target analyte, and a detection probe that can bind the target at a different epitope to generate a visual or colorimetric readout. Our bioinformatics analysis indicated that the selected aptamers, Apt1 and Apt2, bind to distinct, non‐overlapping epitopes on the PEDV‐N protein, enabling their use in a sandwich assay configuration. Due to relatively higher binding affinity, we immobilized the DNA‐Net_Apt2_ construct on the test line as the capture probe via biotin–streptavidin chemistry, while DNA‐Net_Apt1_ was conjugated to gold nanoshell particles (AuNS‐Net_Apt1_) to serve as the reporter or detector probe (Scheme 1). Commercially available streptavidin‐coated gold nanoshells (SA‐AuNS) were chosen due to their high optical density, which enhances readout contrast and improves assay sensitivity [50, 51].

To confirm successful conjugation of DNA‐Net_Apt1_ to the AuNS particles, we employed dynamic light scattering (DLS) to measure hydrodynamic diameter and zeta potential. DLS analysis revealed a substantial increase in particle size upon DNA‐Net conjugation, from 164.40 ± 3.99 nm for unmodified AuNS to 262.24 ± 4.09 nm for AuNS‐Net_Apt1_ (Figure S4a), which suggests the effective surface coverage of AuNS by the DNA‐Net construct. This ∼62% increase in hydrodynamic diameter aligns with the theoretical dimensions of the DNA‐Net and may reflect variation in its spatial orientation on the AuNS surface. Furthermore, the zeta potential decreased from −15.32 ± 1.01 mV for unmodified AuNS to −36.22 ± 1.78 mV following DNA‐Net attachment (Figure S4b), indicating enhanced colloidal stability. This increase in negative surface charge promotes electrostatic repulsion between particles, which is critical for preventing nanoparticle aggregation under high‐ionic‐strength assay conditions, thereby ensuring robust performance of the conjugated assay [52]. Collectively, these results confirm the successful synthesis of the AuNS‐Net_Apt1_ reporter complex, which was subsequently utilized in the LFA platform to enable rapid, sensitive, and specific detection of PEDV‐N protein target.

### 2.5 Analytical Performance Evaluation of the LFA Device Using Recombinant PEDV‐N Protein

**FIGURE 3:**
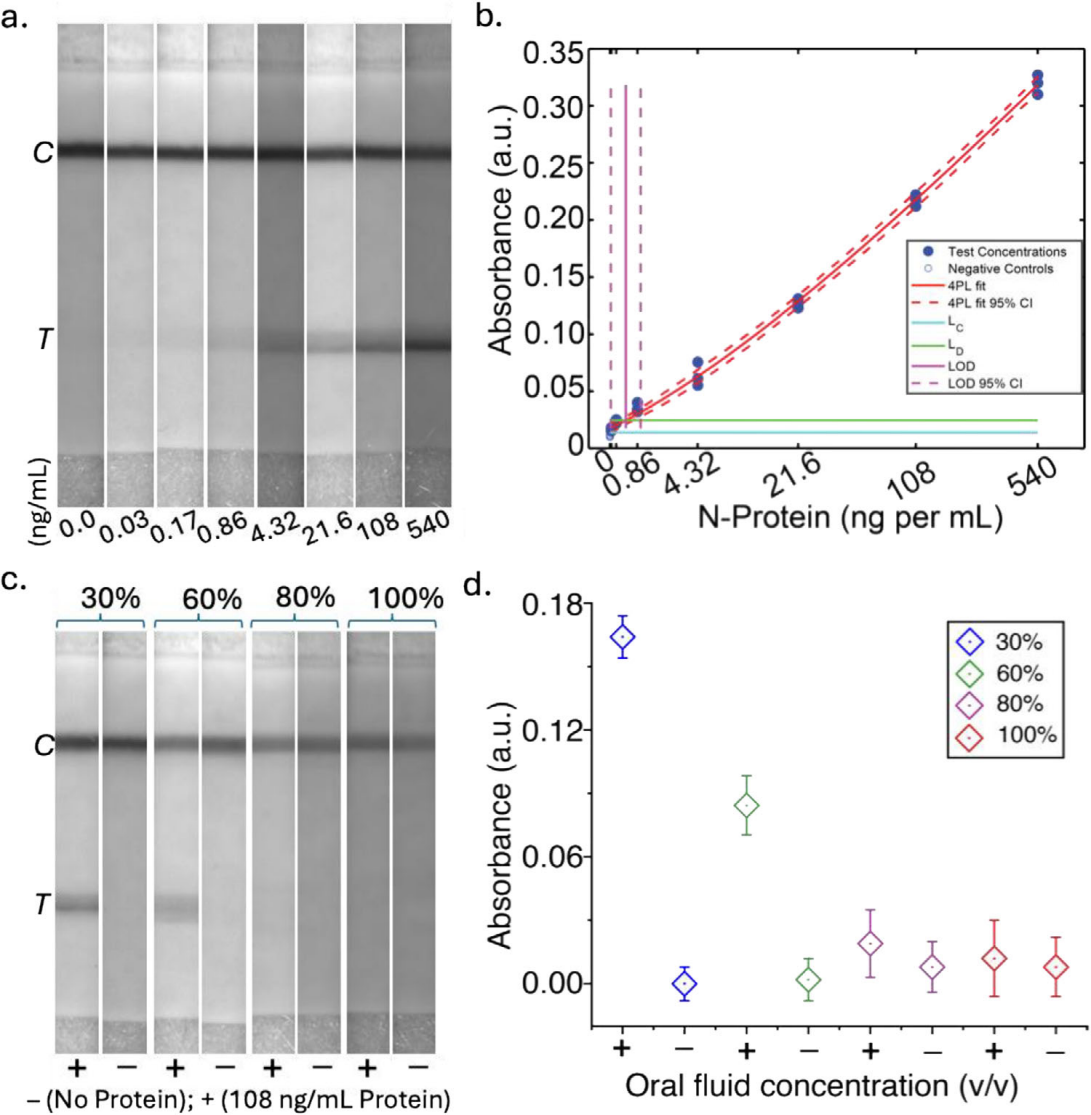
Performance evaluation of the lateral flow assay (LFA) device using recombinant PEDV‐N protein as the target and assessment of its response in varying concentrations of oral fluid. (a) The LFA device was tested with a range of N protein concentrations (0.03–540 ng/mL), showing a concentration‐dependent increase in test line intensity. (b) Absorbance values corresponding to the test line signals were quantified and plotted against N protein concentrations to evaluate the device's sensitivity. 4PL: four‐parameter logistic model; CI: confidence interval; L_C_ and L_D_: limits of blank and detection in the signal domain; LOD: limit of detection in the concentration domain. (c) The assay was tested in oral fluid‐based matrices with oral fluid concentrations ranging from 30% to 100% (v/v). The negative control (−) indicates the absence of N protein, while the positive control (+) represents samples spiked with 108 ng/mL recombinant N protein. (d) Test line absorbance values were quantified and plotted against oral fluid concentration to assess the impact of sample composition on device sensitivity and signal retention under biologically relevant conditions. Data are presented as mean ± SD (standard deviation), with *n* = 3 biologically independent samples.

For downstream LFA development, we prepared nitrocellulose membranes by striping 10 nm of DNA‐Net_Apt2_ constructs onto the test line and 0.25 mg per mL of recombinant PEDV‐N protein onto the control line, respectively (Scheme 1). The recombinant PEDV‐N protein was expressed in the E. coli BL21 (DE3) cells and purified via Ni‐NTA affinity chromatography (Figure S5). To evaluate the LFA's performance in detecting clinically relevant concentrations of N protein, we challenged the test strips with a series of N protein solutions ranging from 0.03 to 540 ng/mL. Each test solution was prepared by dissolving the desired concentration of N protein in sample buffer (1 × PBS, pH 7.4) and mixing it with 50 µL of running buffer (1 × PBS, 1% BSA, 3.5 mm MgCl_2_, and 0.05% Tween‐20), along with 7.5 µL of the AuNS‐Net_Apt1_ reporter complex, immediately prior to application onto the sample pad (Figure 3a). Upon loading, the sample‐reporter mixture migrated along the strip via capillary forces, allowing specific interactions with DNA‐Net_Apt2_ immobilized on the test line (*T*) for target capture and with the pre‐striped N protein on the control line (*C*) for procedural validation.

In the presence of the target protein, a sandwich complex is formed on the test line, consisting of DNA‐Net_Apt2_ (capture probe), the N protein (analyte), and AuNS‐Net_Apt1_ (reporter probe). Simultaneously, excess AuNS‐Net_Apt1_ binds to the pre‐immobilized N protein on the control line, serving as an internal control to confirm reagent flow and reporter functionality. The accumulation of reporter particles at both the test and control lines generated high‐contrast gray color signals with a sample‐to‐result time of 10 min, which were recorded using a portable LFA reader, enabling both qualitative visualization and quantitative absorbance analysis. As expected, increasing recombinant PEDV‐N protein concentrations resulted in enhanced signal intensities at the test line, demonstrating efficient, and concentration‐dependent protein capture by the DNA‐Net_Apt2_ probe, with a visually detectable limit as low as 0.17 ng/mL (Figure 3a).

Of note, the diffused test line and non‐uniform nitrocellulose background in the representative strip image are likely driven by membrane wetting and drying dynamics under our running buffer conditions and the timing of image capture. The PBS‐based running buffer containing 1% BSA, 3.5 mm MgCl_2_, and 0.05% Tween‐20 promotes rapid wetting and prolonged local hydration, which can broaden the apparent flow footprint at the test line when imaged while still wet or shortly after the run. We also cannot exclude minor salt crystallization during drying from PBS plus Mg^2+^ as a contributor to the granular appearance. Consistent with this interpretation, replacing the running buffer with ultrapure water produced a visually cleaner membrane (Movie S1), supporting that the background is primarily buffer‐related drying residue rather than an assay or membrane issue.

To rigorously determine the analytical limit of detection (LOD), we employed a previously reported statistically robust method [53] that estimates both the LOD and its associated 95% confidence interval (CI). Specifically, absorbance versus analyte concentration data were fitted to a four‐parameter logistic (4PL) model, allowing precise conversion of absorbance signals into analyte concentrations. The LOD was then calculated from the fitted curve using Equations S1 and S2 provided Methods section. As shown in Figure 3b, the raw absorbance data across N protein concentrations (blue dots) were fitted with 4PL curves (solid red lines). The dashed red lines represent the 95% CI of the fits. The blue horizontal line denotes the limit of blank, while the green horizontal line marks the LOD in the signal domain. The corresponding pink vertical line indicates the calculated LOD in the concentration domain, with dashed pink lines depicting its 95% CI. Based on this analysis, the LOD of the LFA was determined to be 0.46 ng/mL, with a 95% CI ranging from 0.023 to 0.99 ng/mL (Table S5).

### 2.6 Performance Evaluation of the LFA Device in Pig Oral Fluid Samples

To assess the operational robustness of the LFA device under conditions representative of on‐farm testing, we evaluated its performance in the presence of increasing concentrations of oral fluid from non‐infected pigs. Oral fluid was specifically collected from ropes chewed by pigs, yielding secretions that are more similar to serum than to saliva. Hence, it serves as a suitable matrix to mimic field sample conditions for diagnostic evaluation. Recombinant PEDV‐N protein was spiked into running buffer containing the AuNS‐Net_Apt1_ reporter complex and varying concentrations of oral fluid (30%, 60%, 80%, and 100% v/v). The assay maintained high signal generation at the test line when the oral fluid content was 30%, indicating effective formation of the sandwich complex under this condition (Figure 3c,d). However, a marked reduction or complete absence of the test line signal was observed at oral fluid concentrations ≥ 60% (Figure 3c,d). This decline in assay performance at higher oral fluid concentrations may result from several factors, including increased sample viscosity impeding capillary flow, or nonspecific adsorption of oral fluid components on test strips affecting sandwich complex formation on the test strip. Additional contributors may include nuclease‐mediated degradation of the aptamer and interference from salivary components such as proteins, mucins, and ionic species that can alter binding and surface interactions. Based on these results, we used 30% (v/v) oral fluid as the working matrix for subsequent experiments to preserve robust signal generation while maintaining field‐relevant sample complexity. From a practical standpoint, moderate dilution of oral fluid is common in LFA workflows to balance matrix effects against sensitivity, which is consistent with common practices in rapid antigen testing, where dilution buffers are routinely used to reduce sample viscosity and minimize nonspecific binding [20, 21, 31].

### 2.7 Field‐Relevant Analytical Validation of the LFA Device

**FIGURE 4:**
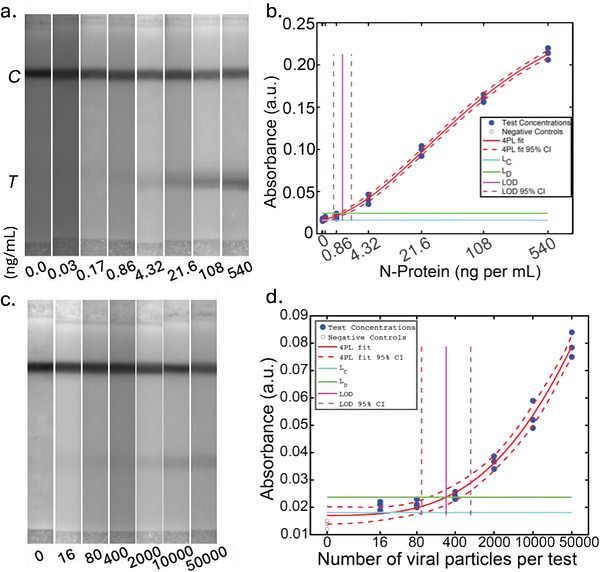
Analytical performance of the lateral flow assay device in a 30% (v/v) porcine oral fluid environment. (a) The device was assessed using recombinant PEDV‐N protein across a concentration range of 0.03–540 ng/mL in running buffer containing 30% oral fluid, demonstrating a clear concentration‐dependent increase in test line intensity. (b) Quantified absorbance values from the test lines were plotted against N protein concentrations to determine assay sensitivity. 4PL: four‐parameter logistic model; CI: confidence interval; L_C_ and L_D_: limits of blank and detection in the signal domain; LOD: limit of detection in the concentration domain. (c) The assay was further tested with wild‐type PEDV virions lysed with 0.1% Triton X‐100. Viruses were serially diluted in 1 × PBS containing 30% porcine oral fluid. A buffer composed of 1 × PBS with 30% oral fluid served as the negative control (0 virus). (d) Absorbance values from each test line were plotted against the total number of viral particles per test to assess the device's performance with the wild‐type virus. Data represent mean ± SD (standard deviation) from *n* = 3 biologically independent replicates.

To ensure assay robustness and reproducibility under near‐field conditions representative of on‐site farm diagnostics, we evaluated the analytical performance of the LFA device in a physiologically relevant matrix consisting of 30% (v/v) pig oral fluid. Using a similar testing procedure, we assessed a range of PEDV‐N protein concentrations from 0.03 to 540 ng/mL, spiked into 30% (v/v) oral fluid prepared in running buffer (Figure 4a). The results demonstrated a concentration‐dependent increase in test line signal intensity, indicating effective capture of the N protein even in the presence of 30% oral fluid. Based on statistical analysis, the LOD was estimated to be 1.33 ng/mL, with a 95% confidence interval (CI) ranging from 0.67 ng/mL (lower limit) to 2.14 ng/mL (upper limit) (Figure 4b and Table S5). The low LOD achieved by the LFA strips is particularly beneficial, as prior studies have demonstrated that oral fluids contain substantially higher levels of viral shedding compared to rectal swabs during the initial 14 days of PEDV infection [10, 54, 55].

Next, we evaluated the device performance against PEDV virions cultured under laboratory conditions using the wild‐type PEDV strain KS1309. The viral stock was lysed by incubating with 0.1% Triton X‐100 for 2–3 min to release viral N proteins [56]. Subsequently, a fivefold serial dilution of the lysed virus was prepared in running buffer containing 30% porcine oral fluid in a 96‐well plate. To each well, 7.5 µL of the AuNS‐Net_Apt1_ reporter complex was added, bringing the total reaction volume to 50 µL. The LFA strips were then subjected to these samples, and a gradual intensification of the gray color signals was observed on both the test and control lines, correlating with increasing viral copy numbers ranging from 16 to 50 000 total viral copies per reaction (Figure 4c). Using a statistically rigorous approach to determine the analytical LOD, the assay demonstrated an LOD of 274.24 viral copies per reaction, with a 95% CI ranging from 97.99 (lower limit) to 761.16 (upper limit) viral copies per reaction (Figure 4d and Table S5). This detection limit represents a significant improvement compared to previously reported PEDV diagnostic methods and commercially available antigen tests, which typically exhibit detection limits in the range of 10^4^ to 10^5^ TCID_50_/mL [21, 22, 23, 24]. The ability to detect such ultralow viral loads highlights the high analytical sensitivity of the DNA‐Net‐enabled LFA platform for PEDV detection in field‐relevant matrices.

### 2.8 Assay Specificity and Robustness Assessment, Including Stability, Repeatability, and Batch‐to‐Batch Reproducibility

**FIGURE 5:**
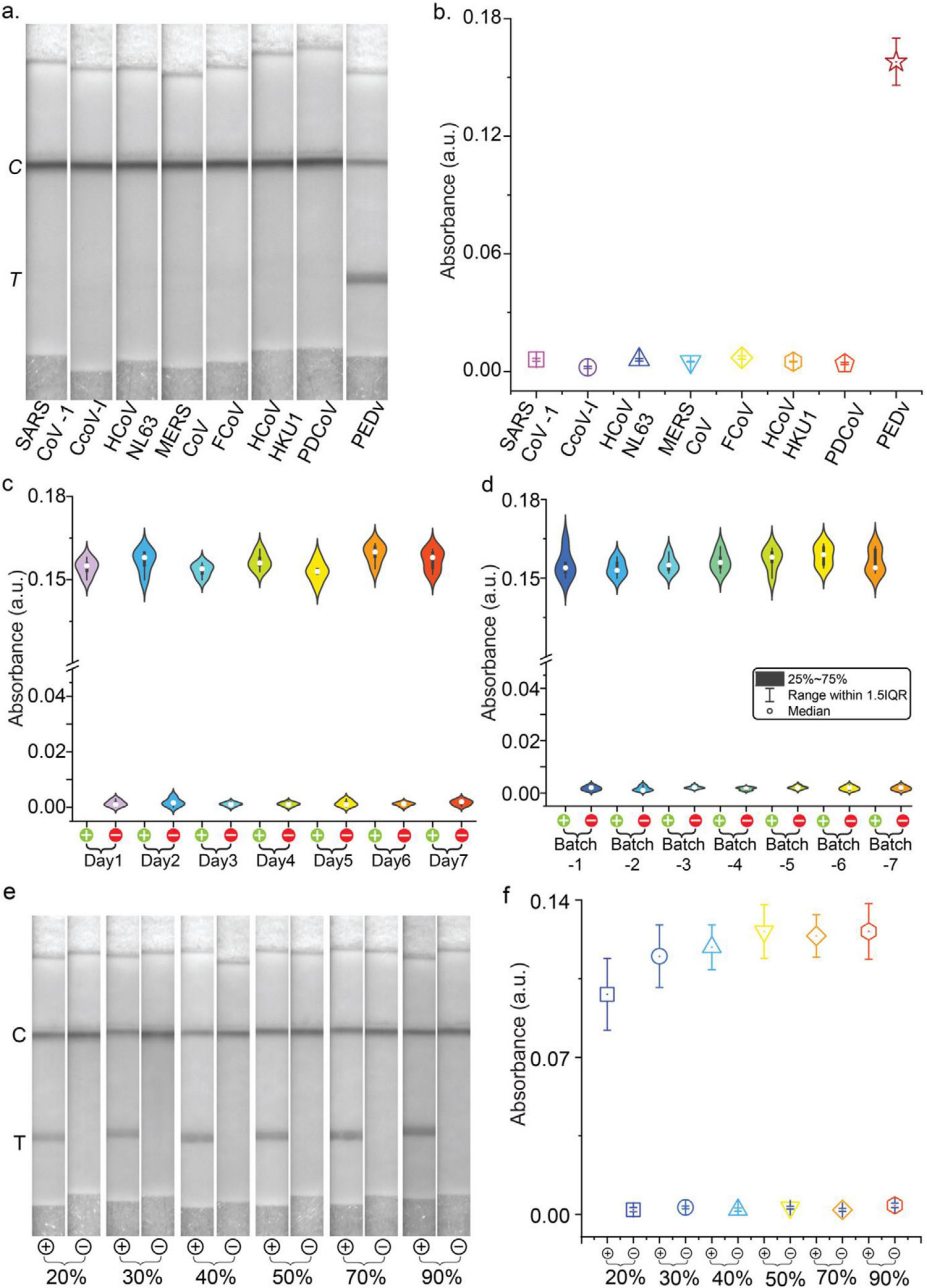
Assay specificity, repeatability, batch‐to‐batch reproducibility, and relative humidity tolerance of the PEDV lateral flow assay. (a) We evaluated assay specificity using a panel of related coronavirus N proteins, including SARS‐CoV‐1 (severe acute respiratory syndrome coronavirus), CCoV I (canine coronavirus), HCoV NL63 (human coronavirus), MERS‐CoV (Middle East respiratory syndrome coronavirus), FCoV (feline coronavirus), HCoV HKU1 (human coronavirus), and PDCoV (porcine deltacoronavirus), alongside the target PEDV. Representative test strip images are shown, where C and T denote the control and test lines. (b) Corresponding quantification of test line absorbance for each condition, showing a strong signal for PEDV and baseline level responses for nontarget viruses. (c) Repeatability assessment of the LFA tests across seven consecutive days, summarized as violin plots of absorbance distributions for positive and negative samples. (d) Reproducibility assessment across independently prepared AuNS–DNA‐Net conjugate batches, summarized as violin plots of absorbance distributions for positive and negative samples across Batches 1–7. For panels c–f, the plus symbol denotes 108 ng per mL N protein spiked in 30% porcine oral fluid, whereas the minus symbol denotes the matched matrix with no protein added. In panels c and d, the center marker denotes the median, the box indicates the 25% to 75% interval, and whiskers represent the range within 1.5 times the interquartile range (1.5 IQR). Data represent mean ± SD from *n* = 5 biologically independent replicates for the day‐to‐day repeatability study and from *n* = 3 biologically independent replicates for the batch‐to‐batch reproducibility study. (e) Representative test strip images demonstrating assay performance across a range of relative humidity conditions from 10% to 90% in the presence (21.6 ng/mL, shown as + symbol) and absence (0 ng/mL, shown as ‐ symbol) of N protein. (f) Quantified test line absorbance for the relative humidity series shown in panel (e). (e‐f) Data represent mean ± SD from *n* = 3 biologically independent replicates.

To evaluate analytical specificity and potential cross reactivity, we challenged the assay with a panel of related coronavirus N proteins from SARS‐CoV‐1 (severe acute respiratory syndrome coronavirus), CCoV I (canine coronavirus), HCoV NL63 (human coronavirus), MERS CoV (Middle East respiratory syndrome coronavirus), FCoV (feline coronavirus), HCoV HKU1 (human coronavirus), and PDCoV (porcine deltacoronavirus) [57]. Each N protein was tested at 108 ng/mL in a matrix containing 30% porcine oral fluid, with PEDV‐N protein included as the positive control (Figure 5a). The LFA device exhibited dark gray colored test line signals only in the presence of the PEDV‐N protein, whereas minimal to no signal was detected for the non‐target N proteins, suggesting the high specificity of our PEDV LFA test. (Figure 5b).

We next assessed repeatability of the PEDV LFA by quantifying within‐day (intra‐assay) and day‐to‐day (inter‐assay) precision using the coefficient of variation (CV) [58]. Unless otherwise mentioned, positive samples consisted of PEDV‐N protein spiked into 30% porcine oral fluid (plus symbol), and the matched matrix without added protein served as the negative control (minus symbol). For each day, five biologically independent replicate strips were tested (*n* = 5). Intra‐assay CV was computed from replicate measurements collected on the same day, whereas inter‐assay precision was evaluated across seven separate days by calculating variability of the daily mean responses (Figure S6). The absorbance distributions are summarized as violin plots, showing tight clustering of the positive signal around a consistent median across all days, while negative controls remained near baseline with minimal signal changes (Figure 5c). Consistent with these distributions, intra‐assay CV ranged from 1.97% to 2.47%, and the overall inter assay CV was 2.18% (Table S6). Collectively, these low CV values indicate strong repeatability and stable signal generation under the tested conditions.

We next evaluated the reproducibility of the PEDV LFA by quantifying precision across independently prepared AuNS–DNA Net conjugate batches using CV. Seven independent conjugate batches were synthesized and integrated into the LFA workflow (Batches 1–7). For each batch, three biologically independent replicate strips were tested (*n* = 3) (Figure S7). As summarized in Table S7, intra‐assay CV ranged from 2.04% to 2.55% across batches, and the overall inter‐assay CV was 2.49%. Absorbance distributions showed consistent positive signal levels across batches, while negative controls remained near baseline (Figure 5d). Collectively, these results indicate robust batch‐to‐batch reproducibility and stable signal generation across independently prepared AuNS–DNA‐Net conjugate lots, supporting reliable assay performance and scalability.

Because PEDV testing may be performed in swine facilities where ambient humidity can vary substantially, we evaluated assay robustness across a relevant range of relative humidity (RH) conditions. We tested assay performance from 20% to 90% RH using 21.6 ng per mL PEDV‐N protein spiked into 30% porcine oral fluid, with the matched unspiked matrix used as the negative control (Figure 5e). Across this range, the test line signal remained consistent and clearly distinguishable from the negative control for RH at or above 30%, indicating that the assay maintains stable performance under moderate to high humidity conditions (Figure 5f). The modest signal reduction observed at 20% RH is consistent with prior reports that low humidity can reduce assay signal, consistent with humidity‐dependent effects on assay operation [59, 60, 61].

To rigorously assess strip stability under storage conditions relevant to shipping and field deployment, a single production batch of fully assembled LFA strips was divided into three cohorts and stored at 4°C, 25°C, or 37°C. At each storage temperature, strips were retrieved after 1 week, 1 month, and 3 months and evaluated using the same processed PEDV sample workflow described above at the respective environment temperature. Briefly, wild‐type PEDV virions were lysed and serially diluted in oral fluid matrix to generate inputs spanning 16 to 10 000 viral particles per test, with a matched no virus control included. Across all storage conditions, representative strips showed the expected viral dose‐dependent increase in test line intensity alongside the control line, indicating preserved strip function after storage (Figures S8–S10). For each time point, test line absorbance values were fit using a four‐parameter logistic model to estimate LOD. The resulting LOD values showed minimal variation over time at each storage temperature, ranging from 266.73 – 284.51 viral particles per test at 4°C (Figure S8 and Table S8), 256.03–289.44 viral particles per test at 25°C (Figure S9 and Table S9), and 264.55–275.85 viral particles per test at 37°C (Figure S10 and Table S10). Collectively, these results indicate that the assembled strips retain their analytical sensitivity for at least 3 months across refrigerated, room temperature, and elevated temperature storage conditions.

### 2.9 Evaluation of PEDV Detection Performance in Diagnostic Samples From Swine Farms

**FIGURE 6:**
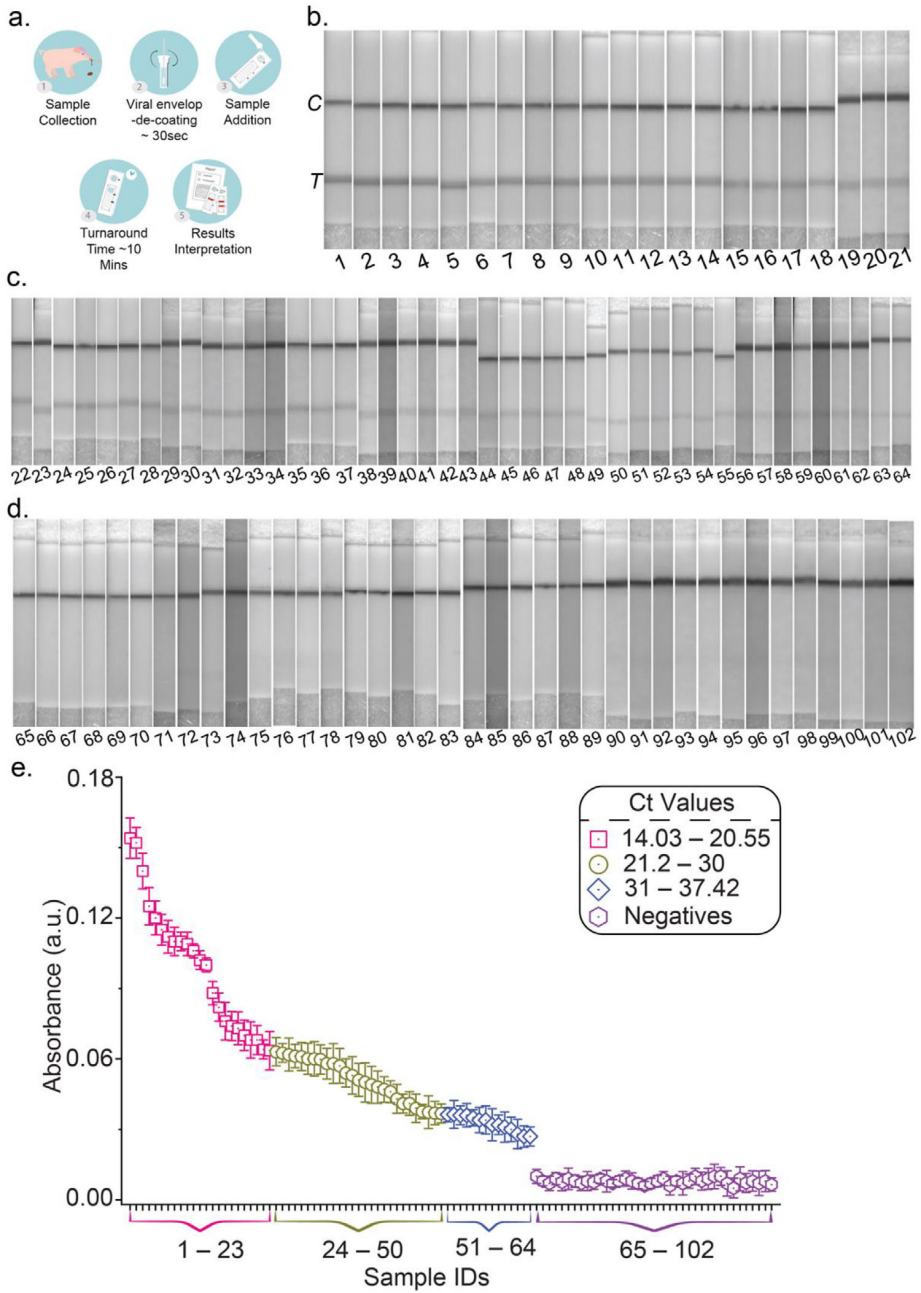
Evaluation of DNA‐Net LFA performance with swine farm samples. (a) Diagnostic workflow and turnaround time for PEDV detection: following sample collection and viral envelope de‐coating process, 50 µL of sample is applied to the LFA device, and results are interpreted after 10 min. (b–d) Representative LFA strips tested with 64 PEDV‐positive samples spanning high to low viral loads (Ct 14–37.42) and 38 PEDV‐negative samples. (e) Corresponding absorbance readings show that high viral load samples (Sample IDs 1–23; Ct values 14–20.55) produced strong test line signals (0.06–0.15), medium viral load samples (IDs 24–50; Ct 21.2–30) showed moderate signals (0.04–0.06), and low viral load samples (IDs 51–64; Ct 31–37.42) exhibited weaker signals (0.03–0.04). Minimal signals (0.003–0.01) were observed for negative samples (IDs 65–102), confirming high assay specificity. Data are presented as mean ± standard deviation from *n* = 3 independent measurements per sample.

Previous studies have reported that PEDV can be detected in rectal swabs, pen fecal samples, and oral fluids by the qRT‐PCR method for up to 6 days post‐exposure (DPE), with viral concentrations found to be higher in pen fecal samples compared to rectal swabs and oral fluids during this period [10]. Beyond 6 DPE, no significant difference was observed in viral concentrations between oral fluids and fecal samples, with detectable viral loads persisting through 69 DPE [10]. Based on these findings, we anticipated that our assay would be capable of detecting PEDV in the diverse clinical sample types available to us. In this study, we analyzed a panel of swine samples received from Veterinary Diagnostic Laboratories (VDL) at the University of Illinois Urbana‐Champaign and Iowa State University. These samples were submitted to VDL from multiple farms with different sample types, including pen fecal swabs, rectal swabs, and oral fluids, covering a broad range of viral loads as indicated by their respective Ct values, which ranged from 14.00 (very high viral load) to 37.42 (very low viral load). A total of 102 PEDV‐related clinical samples were evaluated, comprising 64 samples confirmed as PEDV‐positive and 38 as PEDV‐negative by RT‐PCR (Table S11). While this cohort includes multiple matrices and independent farm submissions, the specimens were predominantly sourced from diagnostic submissions in the US Midwest, reflecting sample availability during the study period. Accordingly, following results support strong agreement with qRT‐PCR within the evaluated field cohort, and larger blinded studies with broader geographic sampling and multi‐laboratory testing will be valuable to further establish generalizability across diverse production systems and outbreak contexts. A viral sample de‐enveloping and dilution, the clinical samples were applied to the LFA device (Figure 6a). The assay produced distinct absorbance signals corresponding to different viral load categories. As shown in Figure 6b,c, high viral load samples (Sample IDs 1–23; Ct values 14–20.55) exhibited strong test line absorbance values ranging from 0.065 to 0.15; medium viral load samples (Sample IDs 24–50; Ct values 21.2–30) showed absorbance values between 0.04 and 0.06; and low viral load samples (Sample IDs 51–64; Ct values 31–37.42) demonstrated absorbance values of 0.03–0.04. In contrast, negative samples (Sample IDs 65–102, and Figure 6d) exhibited minimal absorbance values ranging from 0.003 to 0.01 (Figures 6e), indicating minimal cross‐reactivity and low false‐positive rates.

To evaluate diagnostic performance, clinical metrics including accuracy, sensitivity, specificity, positive predictive value (PPV), and negative predictive value (NPV) were calculated [62]. The LFA device demonstrated an overall diagnostic accuracy of 97.06%, sensitivity of 96.92%, specificity of 97.30%, PPV of 98.44%, and NPV of 94.74%, aligning well within the standard thresholds for reliable diagnostic assays and outperforming several previously reported PEDV diagnostic methods [21, 22, 23, 24, 31, 63]. For example, using the same set of aptamers, Li et al. [31] reported a sensitivity of 83%, specificity of 100%, and accuracy of 92% when testing only 12 clinically derived swine saliva samples, with their lowest detectable Ct value being 33.17 and a sample‐to‐result time of 60 min. In contrast, the DNA‐Net‐enabled LFA platform described here achieved higher sensitivity, the ability to detect samples with Ct ≤ 37.42, and a significantly shorter sample‐to‐result time (∼10 min). It is important to note that such a significant improvement in diagnostic performance arose not from any single functional element alone, but from the way the individual components were integrated into one platform. In the present study, two PEDV‐targeting aptamers with distinct binding profiles were incorporated into a spatially organized DNA‐Net scaffold to promote multivalent target engagement within a sandwich assay format, and this design was directly translated into an LFA device. As a result, the binding advantage conferred by nanoscale multivalent organization could be converted into rapid and sensitive detection in clinically relevant samples. This distinguishes the current study from prior aptamer‐based assays that did not incorporate multivalent DNA nanostructure organization in an LFA format [31], and also from earlier DNA nanostructure‐based sensing studies that were not validated in a field‐relevant diagnostic settings [35, 39]. Together, these results highlight the value of coupling programmable nanoscale ligand organization with a practical point‐of‐care assay format for real‐world PEDV diagnostics.

### 2.10 Cost and Long‐Term Stability Considerations for the Rapid Test Development

The total cost to manufacture the proposed DNA Net‐based rapid test is approximately 0.8 to 1.5 USD per test (Table S12). Several practical pathways can further reduce per‐test cost as the platform transitions from lab settings to manufacturing scale. First, the DNA‐Net construct is well‐suited for low‐cost production because it relies on a limited set of short synthetic DNA oligonucleotides (*n* = 66) that are unmodified (except one biotin strand) and can be produced by standard chemistry. Second, DNA‐Net self‐assembly can be achieved by one‐pot thermal annealing in aqueous buffer, followed by a single‐step conjugation to gold nanoparticles. These steps can be scaled up to generate large batches of DNA‐Net gold conjugate that are then sprayed across large batches of LFA strip, thereby driving the incremental DNA‐Net cost per strip to a small fraction of the total bill of materials. Additional cost reductions can be realized through manufacturing decisions that maximize economies of scale. These include specifying desalting as the default purification method where high purity is not required for DNA nanostructure assembly and purchasing oligonucleotides on a large scale. Importantly, annealing and conjugation are readily automatable and compatible with established workflows used for antibody gold conjugate, which further reduces handling steps. Finally, the remaining assay components leverage established low‐cost LFA components, including standard membranes, gold nanoparticles, and simple buffer formulations that are compatible with drying or lyophilization for ambient storage.

From a translational perspective, the long‐term stability of the DNA‐Net LFA is governed primarily by two molecular factors: (i) integrity of the DNA aptamers and scaffold strands, and (ii) preservation of the assembled DNA‐Net architecture under storage and use conditions. DNA aptamers are intrinsically more robust than proteins to temperature excursions and pH changes, and in the dry state used in LFA strips they exhibit excellent shelf stability [64, 65]. The main risk is nuclease‐mediated degradation, which is largely associated with residual biological contaminants [66]. This risk is minimized in practice by using nuclease‐free reagents and maintaining clean manufacturing conditions. Previous work on DNA nanostructures has further shown that appropriate formulation (for example, inclusion of sugars [67], crowding agents [68], ionic liquids [69]) can markedly enhance resistance to nuclease attack and maintain structural integrity over extended storage, providing an additional layer of confidence that multistrand architectures such as the DNA‐Net can be stabilized for practical use. In addition, packaging each strip in an individual sealed foil pouch with desiccant to control humidity and oxygen exposure is expected to further increase shelf life, consistent with established practices in commercial lateral flow manufacturing [70, 71].

## 3 Conclusion

Despite decades of research and the availability of sensitive molecular diagnostic tools, effective on‐farm management of PEDV outbreaks remains hindered by the lack of rapid, sensitive, and easy‐to‐use detection methods deployable directly at the point of need. To address this gap, we developed a multivalent DNA nanostructure‐enabled lateral flow assay for the ultrasensitive and field‐deployable detection of PEDV. By leveraging the programmable assembly of net‐shaped DNA nanostructures (DNA‐Net), we spatially organized PEDV‐specific aptamers into multivalent configurations, resulting in a remarkable ∼1000‐fold enhancement in binding avidity compared to free monomeric aptamers. When implemented in an LFA format, the DNA‐Net_Apt_ constructs achieved detection limits of ∼1.33 ng/mL for recombinant PEDV nucleocapsid protein and ∼278 viral copies per test for cultured wild‐type virus in a natural pig oral fluid matrix. In a cohort of 102 diagnostic samples collected from multiple swine farms, the assay reached an overall diagnostic accuracy of 97.06%, with a sensitivity of 96.92%, a specificity of 97.30%, a PPV of 98.44%, and an NPV of 94.74% relative to qRT‐PCR. Notably, the assay was capable of detecting ultra‐low viral loads corresponding to Ct values ≤ 37.42 and delivered results within 10 min, without the need for complex instrumentation, nucleic acid extraction, or amplification steps. Furthermore, since the clinical specimens were primarily obtained from diagnostic submissions in the US Midwest, future work will focus on larger blinded multi‐region validation studies, ideally including inter‐laboratory testing, to further confirm performance across geographically diverse farm systems.

Overall, these findings underscore the potential of integrating designer DNA nanostructures with aptamer‐based recognition elements to overcome key limitations of existing diagnostic modalities, particularly in agricultural settings where rapid on‐site testing is essential for effective outbreak control. Compared to conventional RT‐PCR or isothermal amplification‐based assays, the DNA‐Net‐based LFA offers substantial advantages in operational simplicity, assay turnaround time, and field deployability. Moreover, the chemical stability and customizable architecture of aptamer‐tethered DNA nanostructures provide a robust platform suitable for deployment in challenging environments where cold chain maintenance is impractical. Looking ahead, future work will focus on expanding this platform to enable multiplexed detection of PEDV alongside other co‐circulating enteric swine pathogens, such as PDCoV, within a single assay format. Beyond swine health, the modularity and adaptability of the DNA‐Net platform can be extended to a broad range of viral, bacterial, and toxin targets, positioning this technology as a versatile tool for veterinary diagnostics, food safety monitoring, and broader One Health applications.

## Conflicts of Interest

The authors declare the following competing financial interest(s): A U.S. provisional patent has been filed in 2024 to cover the background technology for the study reported in this manuscript.

## Supporting information




**Supporting File 1**: adhm71300‐sup‐0001‐SuppMat.pdf.

**Supporting File 2**: adhm71300‐sup‐0002‐MovieS1.mp4.

**Supporting File 3**: adhm71300‐sup‐0003‐MovieS2.mp4.

**Supporting File 4**: adhm71300‐sup‐0004‐MovieS3.mp4.

## Data Availability

The data that support the findings of this study are available in the supplementary material of this article.
