# Supplementary material for: Ultrasensitive Detection of Porcine Epidemic Diarrhea Virus Infections Using Multivalent DNA Nanostructure‐Enabled Lateral Flow Assay
Source: Adv Healthc Mater. 2026 Jun 23;15(26):e04037. doi: 10.1002/adhm.202504037 (PMC13356504; doi:10.1002/adhm.202504037)
Supplement: Supplementary file 1 — Supporting File 1: adhm71300‐sup‐0001‐SuppMat.pdf. [file ADHM-15-0-s003.pdf]

Supporting Information

Ultrasensitive Detection of Porcine Epidemic Diarrhea Virus Infections Using Multivalent DNA Nanostructure-Enabled Lateral Flow Assay

Saurabh Umrao[1,2,3,4,5,*], Akhila Naru[6], Dhanush Gandavadi[1,2,3], Mengxi Zheng[1,2,3], Saraswathi Lanka[7], Hyeongjun Cho[1,2,3], Chau Nguyen Minh Hoang[1], Jianqiang Zhang[8], Ying Fang[3,6], Xing Wang[1,2,3,4,5,*]

[1] Department of Bioengineering, University of Illinois at Urbana-Champaign, Urbana, IL, USA

[2] Nick Holonyak Jr. Micro and Nanotechnology Laboratory, University of Illinois at Urbana-Champaign, Urbana, IL, USA

[3] Carl R. Woese Institute for Genomic Biology, University of Illinois at Urbana-Champaign, Urbana, IL, USA

[4] Department of Chemistry, University of Illinois at Urbana-Champaign, Urbana, IL, USA

[5] Cancer Center at Illinois, University of Illinois at Urbana-Champaign, Urbana, IL, USA

[6] Department of Pathobiology, College of Veterinary Medicine, University of Illinois at Urbana-Champaign, Urbana, IL, USA

[7] Veterinary Diagnostic Laboratory, College of Veterinary Medicine, University of Illinois at Urbana-Champaign, Urbana, IL, USA

[8] Department of Veterinary Diagnostic and Production Animal Medicine, College of Veterinary Medicine, Iowa State University, Ames, IA, USA

* E-mail:

Saurabh Umrao: usaurabh@illinois.edu

Xing Wang: xingw@illinois.edu

Materials and Methods

Reagents.

DNA aptamers and oligonucleotide strands used for constructing the DNA-Net were synthesized by Integrated DNA Technologies (Coralville, Iowa). The sequences for Apt1 (PEA1-3) and Apt2 (PEA2) were adapted from previous report[1], with additional 3' overhangs (italicized below) incorporated to facilitate their attachment to the DNA-Net constructs. The sequences were as follows:

Apt1-
AGCAGATGACCATTCTGATTGCTTCCTAAGCGGGTTCCCTTTTAGGGGCTTGGG*TTTTGCT TCGTGCAGG*;

Apt2 – CCACGCGCCTTCTCTCTGTCTGGGTTCCCTTCTAGGGTTT*TTTTGCTTCGTGCAGG*.

Streptavidin-functionalized gold nanoshells (SA-AuNS, 150 nm, GSIR150) were sourced from Nanocomposix (San Diego, CA), while streptavidin-modified nitrocellulose membranes (Cat# pro-338) were obtained from ProSpec Bio (US). Reagents required for preparing sample, conjugation, and running buffers including D(+)-Trehalose dihydrate (182551000), D(+)-Biotin (A14207-03), sodium chloride (S9625), magnesium chloride hexahydrate (442611), sucrose (84097), HEPES buffer (J16924-K2, Thermo Scientific), Tween-20 (5075031), bovine serum albumin (BSA, BAH64), human serum (H5667), and phosphate-buffered saline (PBS, AM9625) were purchased from MilliporeSigma (St. Louis, MO).

The ALFRD Lateral Flow Reagent Dispensing machine (part number: 07.711.01) was acquired from Claremont Biosolutions (CA). Paper-based components of the lateral flow assay, including nitrocellulose membranes (VIVID 120 LFNC), absorption pad (CF5), conjugate pads (8980), and laminated backing card (HF000MC100), were purchased from Pall Corporation, Cytiva, Ahlstrom (Helsinki, Finland), and Millipore Sigma respectively. For precise cutting of these components, a high-speed Rapid Test Strip Cutter (GQ1010d) from Werfen Automation (China) was used. Gastight glass syringes (1725TLL, 250 μL) were also obtained from MilliporeSigma (St. Louis, MO).

Preparation and Assembly of LFA Test Strips

The LFA device was assembled using a conjugation pad, nitrocellulose membrane (NCM), and absorption pad arranged sequentially, with a 2 mm overlap between each component to ensure

continuous capillary flow. Test line conjugates were prepared by mixing 120 nM DNA-Net$_{Apt2}$ with 126 nM nitrocellulose-streptavidin in 1× TAE-Mg^{2+}-K^+ buffer, with a total volume of 200 µL. This mixture was incubated at room temperature for 45 minutes on a rotator prior to printing. For the control line, a solution of recombinant PEDV-N protein at a concentration of 0.25 mg/mL was used. Both the test line and control line reagents were dispensed onto their respective positions on the NCM using the reagent dispensing machine at a flow rate of 30 µL/min. Following reagent deposition, all components were assembled onto backing cards and cut into individual strips measuring 60 mm × 4 mm using a test strip cutting machine. The prepared strips were then stored in aluminum foil pouches with desiccant bags to maintain dryness and ensure storage stability.

Signal Detection and Quantification

Each LFA device was immersed into individual wells of a 96-well plate (part number: 10062-900, VWR) containing 50 µL of total solution. This solution comprised 1X phosphate-buffered saline (PBS) supplemented with 1% BSA, potassium chloride, magnesium chloride, 7.5 µL of the reporter particle suspension, and the target sample at the desired concentration.

Signal generation at the test line was amplified by the reporter construct and subsequently captured using a portable reader (Lumos Leelu, Model# LUMOS-V3-03, Lumos Diagnostics Inc.). The reader was equipped with an integrated LED light source emitting red illumination optimized for gold nanoshells (AuNS) and operated with an exposure time of 3 ms, enabling precise imaging of the test strip. The AuNS reporter particles absorbed part of the incident light, resulting in a corresponding increase in reflected light intensity from the test line region on the nitrocellulose membrane. For quantification, background correction was performed by calculating the average pixel intensity values from two background regions located to the left and right of the test line peak intensity. After background subtraction, the absorbed light was quantified and reported as the final signal.

Limit of detection calculation.

The analytical limit of detection (LOD) was estimated using the four-parameter logistic (4PL) framework described by Holstein et al.[2] and implemented in MATLAB. Calibration data were fit by nonlinear regression to the 4PL model **(Equation 1)**, where y denotes the measured assay response, a and d are the upper and lower asymptotes, c is the inflection point, and b is the slope parameter. To enable inclusion of the blank in the regression, concentrations were expressed on a transformed axis $x = \log_{10}(C + 2)$, where C is the analyte concentration. A signal decision

threshold L_D was determined using mathematical model reported by Holstein et al.[2] and the corresponding concentration LOD was obtained by analytical inversion of the fitted 4PL curve **(Equation 2)**. Unless otherwise noted, parameter estimation and associated calculations followed the published MATLAB implementation.[2] Parameter values obtained under the experimental conditions used in this study are summarized in **Tables S4-S5, and S8–S10**.

$$y = \frac{a-d}{1+\left(\frac{x}{c}\right)^b} + d \qquad (1)$$

$$Log\ (LOD+2) = c\left[\left(\frac{a-d}{L_D-d} - 1\right)\right]^{1/b} \qquad (2)$$

Clinical Evaluation of the LFA device.

Clinical sensitivity, specificity, and diagnostic accuracy were calculated as:

Sensitivity = True Positive ÷ (True Positive + False Negative)

Specificity = True Negative ÷ (True Negative + False Positive)

Accuracy= (True Positive + True Negative) ÷ (True Positive + True Negative + False Positive + False Negative)

Positive Predictive Value (PPV) = True Positive ÷ (True Positive + False Positive)

Negative Predictive Value (NPV) = True Negative ÷ (True Negative + False Negative)

Cells

Vero76 (ATCC, CRL-1587 ™) cells were grown in Eagle's minimum essential medium (MEM) (Invitrogen; Waltham, MA) containing 10% fetal bovine serum (FBS) (Sigma; St. Louis, MO), and 1% Penicillin-Streptomycin (Gibco; Waltham, MA).

Virus Preparation

PEDV strain KS1309 (GenBank Accession: KJ184549.1) was propagated in Vero76 cells. Cells were infected at a multiplicity of infection (MOI) of 0.01 in MEM infection medium with 2% Trptose Phosphate Broth solution (Sigma), 1ug/ml L-1-Tosylamide-2-phenylethyl chloromethyl ketone (TPCK) (Sigma). The cell culture supernatant was collected at 72 hours post-infection (hpi).

Quantitative Real-Time PCR (qPCR) for PEDV Detection

Viral RNA was isolated from cell culture supernatant using the QIAamp Viral RNA Mini Kit (Qiagen). A quantitative real-time PCR (qPCR) was performed using the EZ-PED/TGE/PDCoV MPX 1.1 Master Mix and Enzyme kit (SKU: TC-9083-192; Tetracore Inc., Rockville, MD) according to the manufacturer's protocol. While the lysis buffer contained an internal inhibition control (IC), we were able to monitor the efficiency and consistency of RNA extraction. A RT-qPCR reaction has a final volume for the whole PCR reaction of 25 µL and has 18 µL of ready-to-use Master Mix (containing enzyme) and 7 µL of extracted viral RNA with IC. The PCR run included a negative extraction control, individual positive controls for PEDV, a no-template control. Amplification was completed using the Applied Biosystems Quant Studio under the following cycling conditions: cDNA reverse transcription at 48 °C for 15 minutes; initial denaturation at 95 °C for 2 minutes, followed by 45 cycles of denaturation at 95 °C for 5 seconds and annealing/extension at 60 °C for 40 seconds. Data was analyzed by Design & Analysis Software 2.8.0 (Thermo Fisher Scientific), and samples with a cycle threshold (Ct) greater than 40 were considered negative.

Quantitation of PEDV RNA by Real-Time RT-PCR

Quantitative real-time PCR of PEDV virus was performed using the EZ-PED/TGE/PDCoV MPX 1.1 RT-PCR kit that provided an excellent positive signal with a mean cycle threshold (Ct) value of 21.839 for PEDV. The corresponding estimate for standard curve values using the Ct suggested approximately 1.67×10^{10} copies/mL which indicated a very high concentration of PEDv RNA from cell culture supernatant.

Recombinant Protein Expression and Purification

Viral RNA was extracted using a QIAamp Viral RNA Mini Kit (Qiagen) and cDNA was created for PEDV using SuperScript® III One-Step RT-PCR System with Platinum® Taq DNA polymerase. Primers were used to amplify the N gene of the Porcine Epidemic Diarrhea Virus (PEDV). After PCR was performed, the fragments were sub-cloned into the pET28α (+) vector (Novagen, 6×His tag), followed by transformation into E. coli BL21 (DE3) (Novagen, Madison, WI) for protein expression. The overnight culture was grown at 37°C in 10 mL LB medium (50 µg/mL kanamycin) and used to inoculate 250 mL 2× YT media (kanamycin). The culture was allowed to grow for 3 hours (OD_{600} = 0.5-0.7) before being induced with 0.1 mM IPTG and allowed to grow for an additional 4 hours. Cells were harvested via centrifugation (10,000 × g for 15 minutes at 4°C). The final pellet was resuspended in B-PER™ reagent and sonicated. The lysate was agitated for

10 min at 4 °C and then centrifuged at 15,000 × g for 10 min to obtain the supernatant, which was treated at 4 °C overnight with Ni-NTA agarose beads (QIAgen, Chatsworth, CA) to purify the His-tagged N protein. After protein capture on the beads, the protein was eluted using the buffer containing 50 mM $NaH_2PO_4·2H_2O$, 200 mM imidazole, 300 mM NaCl, pH 8.0 using the Model 422 Electro-Eluter (Bio-Rad; Hercules, CA). The eluted proteins were dialyzed three times in 1× PBS at 4 °C. Results from SDS-PAGE showed a prominent band at the expected size of His-tagged N protein (54.94 kDa) confirming successful expression and purification **(Figure S5A)**. To verify the identity of the expressed protein, a Western blot was performed using mouse monoclonal antibody against PEDV-N protein. A prominent band appeared at the same calculated molecular weight (54.94 kDa) confirming the presence of recombinant N protein, and its antigenic specificity **(Figure S5B)**.

SDS PAGE and Western blot

Recombinant proteins were mixed with 4× Laemmli sample buffer and heated for 5 minutes at 95 °C. Proteins were separated on SDS-PAGE gels and transferred to nitrocellulose membrane. Membranes were blocked in 5% skim milk, made in PBS, overnight at 4 °C. After blocking, membranes were incubated at room temperature with PEDV N monoclonal antibody for 1 hour. After washing membranes with PBST (1× PBS with 0.05% Tween 20), membranes were incubated with IRDye® 800CW Goat anti-Mouse IgG (H + L) secondary antibody (LI-COR Biosciences, Lincoln, NE) for 1 hour at room temperature. After washing membranes with PBST, fluorescence was read using the Odyssey® Infrared Imaging System (LI-COR Biosciences).

One-Pot Assembly of DNA-Net Constructs.

DNA-Net constructs were synthesized using a well-established protocol described previously[3]. Briefly, DNA-Net constructs were prepared in a one-pot reaction by mixing all component strands at a final concentration of 200 nM in 1× TAE-Mg^{2+}-K^+ buffer (pH 7.5). The buffer comprised 40 mM Tris-acetate, 2 mM EDTA, 12.5 mM magnesium acetate, and 10 mM potassium chloride. To assemble the DNA-Net, DNA aptamers and component DNA strands were combined at a 27:1 stoichiometric ratio, reflecting the 27 aptamer docking sites present on the nanostructure. Two distinct DNA-Net complexes were prepared using Apt1 and Apt2 aptamers separately, resulting in: DNA-Net_{Apt1}, and DNA-Net_{Apt2} constructs. The reaction mixtures were subjected to a thermal annealing process by gradually cooling from 90 °C to 23 °C over 14 hours using a thermal cycler (TProfessional TRIO PCR Thermocycler). Following annealing, the assembled DNA-Net constructs were stored at 4°C to maintain structural stability prior to their use in assays.

Validation of DNA-Net Assembly by Agarose Gel Electrophoresis.

To confirm the successful assembly of the DNA-Net construct, agarose gel electrophoresis (AGE) was performed. A 1% agarose gel was prepared using 1× TAE-Mg^{2+} buffer, which contained 40 mM Tris-acetate, 2 mM EDTA, and 12.5 mM magnesium acetate (pH 7.5). The 1 kb+ DNA ladder (SM1333, Thermo Scientific) was used as a molecular weight marker to compare the migration behavior of the DNA-Net construct with known DNA fragment sizes. Electrophoresis was carried out at a constant voltage of 60 V for 2–3 hours. Following separation, the gels were stained with SYBR Green (Thermo Fisher) to visualize the DNA bands and subsequently imaged using a GelDoc imaging system (Bio-Rad).

Atomic Force Microscopy (AFM) imaging.

For AFM imaging, a 5 µL aliquot of the buffer solution containing 2 nM DNA-Net was deposited onto a freshly cleaved mica surface (Ted Pella Inc.) and incubated for 5 minutes. Following incubation, the sample was rinsed by adding 1× TAE-Mg^{2+} buffer, and the solution was immediately removed using a pipette. Subsequently, 80 µL of fresh 1× TAE-Mg^{2+} buffer was added to the mica surface to maintain hydration during imaging. AFM imaging was performed in tapping mode under fluid conditions using an Asylum AFM system equipped with an MSNL-10 probe (Bruker Nano, Inc.).

Synthesis of reporter assay.

To prepare the reporter assay construct (AuNS-Net_{Apt1}), the DNA-Net_{Apt1} was conjugated to 150 nm streptavidin-modified gold nanoshells (SA-AuNS) using biotin-streptavidin chemistry. The SA-AuNS solution was first vortexed vigorously for 2 minutes to ensure homogeneous dispersion. In a separate tube, the SA-AuNS solution, conjugation buffer, and DNA-Net_{Apt1} solution were combined in a 5:4:1 volume ratio. The mixture was incubated at ambient temperature for 30 minutes with continuous rotation to facilitate efficient binding of the DNA-Net constructs to the AuNS surface. To terminate the reaction and block any remaining unreacted streptavidin sites, excess biotin was added to the mixture. The resulting reporter construct (AuNS-Net_{Apt1}) was then incubated at room temperature for an additional 15 minutes under continuous rotation to ensure complete blocking and stabilization of the conjugate.

Dynamic Light Scattering and Zeta Potential Measurements.

Hydrodynamic diameter and zeta potential measurements were conducted using a Malvern Zetasizer Nano ZS90 instrument (Malvern Panalytical Ltd., Malvern, UK) equipped with a backscattering detector operating at 173°. Samples were analyzed in disposable polystyrene cuvettes (DTS0012) using the ZEN1002 Universal Dip Cell for zeta potential measurements. For optimal measurement conditions, the AuNS stock solution was diluted 10^6-fold in 0.02 µm filtered PBS buffer. Zeta potential, which quantifies the electrostatic potential at the interface between the nanoparticle surface and the bulk medium, provides insights into particle surface charge and colloidal stability. To mitigate potential artifacts from Joule heating and polarization, a 60-second equilibration delay was applied between consecutive zeta potential measurements. All measurements were performed at 25°C, consisting of five sequential zeta potential readings followed by five hydrodynamic diameter measurements using backscatter detection to ensure accuracy and reproducibility.

Surface Plasmon Resonance (SPR) Methodology.

Surface Plasmon Resonance (SPR) analysis was performed to determine the binding kinetics of the constructs. Wild-type recombinant PEDV-N protein was immobilized onto flow cell (FC2) of a research-grade CM5 S-Series SPR chip (GE Healthcare, Uppsala, Sweden) using a standard amine coupling protocol. Briefly, the carboxymethyl groups on flow cells 1 and 2 of the CM5 chip surface were activated by injecting a freshly prepared 1:1 mixture of N-ethyl-N-(dimethylaminopropyl) carbodiimide (EDC) and N-hydroxysuccinimide (NHS) (final concentration of 50 mM each) for 420 seconds at a flow rate of 5 µL/min. Flow Cell 1 was used as a reference cell with no protein immobilization, while flow Cell 2 was used as a sample cell by injecting a 50 µg/mL solution of recombinant PEDV-N protein in 10 mM sodium acetate buffer (pH 5.5), yielding a baseline increase of ~1683 RU, indicating successful protein immobilization. Unreacted carboxymethyl groups on both flow cells were blocked by injecting 1 M ethanolamine for 600 seconds at 5 µL/min. Analyte solutions, including threefold serial dilutions of free DNA aptamers, DNA-Net constructs without aptamers (control), and DNA-Net constructs functionalized with different aptamers, were prepared and injected over the sensor chip at a flow rate of 5 µL/min. The running buffer consisted of 1× TAE-Mg^{2+}-K^+ buffer (pH 7.5), containing 40 mM Tris-acetate, 2 mM EDTA, 12.5 mM magnesium acetate, and 10 mM potassium chloride. Following analyte injection, dissociation was monitored by flowing running buffer over the sensor surface for 5 minutes. SPR response (sensorgrams) was recorded as a function of time at 25°C using a BIAcore T200 instrument (GE Healthcare, Sweden) operated with BIAcore T200 control software. All measurements were performed in triplicate. The resulting sensorgrams were analyzed to

determine the kinetic parameters, including association rate constant (k_a), dissociation rate constant (k_d), and equilibrium dissociation constant (K_D, calculated as k_d/k_a), by locally fitting the association and dissociation phases using BiaEvaluation software 4.0.1 (GE Healthcare, Sweden).

Sequential sandwich binding was evaluated using a capture format on a Series S Sensor Chip SA (Cat. 29104992, GE Healthcare, Uppsala, Sweden) with pre immobilized streptavidin. Prior to ligand capture, the SA surface was conditioned with three consecutive injections of 1.0 M NaCl in 50 mM NaOH for 60 s each at 10 µL min^{-1} to remove loosely bound material and stabilize the baseline. Folded biotinylated Apt2 was diluted in running buffer and injected over the active flow cell (FC2) until the set immobilization level was reached, while FC1 served as the reference channel. Residual streptavidin binding sites were then blocked by injecting 50 µM free biotin in running buffer to minimize nonspecific binding during subsequent injections. After establishing a stable baseline in running buffer, recombinant PEDV N protein was injected over both flow cells to allow capture by surface immobilized Apt2, followed by a brief running buffer wash to remove unbound protein. Apt1 was subsequently injected to probe secondary binding to the preformed Apt2–N protein complex. Sandwich formation was inferred when an additional increase in resonance units was observed during the Apt1 injection step relative to the preceding wash. As a specificity control, the same sequential injection sequence was performed using an influenza aptamer[2] in place of Apt1, and minimal response in the third injection step was used to confirm sequence specific secondary binding.

To mimic the lateral flow assay configuration, SPR experiments were also performed using biotinylated DNA Net constructs on the Series S Sensor Chip SA. DNA-Net$_{Apt2}$ was captured on FC4 using the same conditioning, immobilization, and biotin blocking workflow described above, with FC3 left blank as the reference. Recombinant PEDV N protein was then injected to bind the immobilized DNA-Net$_{Apt2}$ surface, followed by a brief wash and subsequent injection of DNA-Net$_{Apt1}$ as the secondary binder. Preservation of the sandwich binding mode on the nanostructure was concluded when a pronounced additional resonance unit increase occurred during the DNA-Net$_{Apt1}$ injection step. As a scaffold level control, a DNA-Net construct bearing non-target influenza aptamers were evaluated under the same sequential program, and minimal response throughout the sequence confirmed that the secondary signal required aptamer mediated recognition rather than nonspecific interactions of the DNA-Net scaffold. In all experiments, regeneration of the sensor surface between different injections was achieved with a 30-second pulse of 10 mM

glycine buffer (pH 2.5) at a flow rate of 30 µL min^{-1}, followed by a 5-minute stabilization interval to re-establish baseline conditions.

LFA Migration Kinetics with and without Triton X-100 in the Running Buffer.

To isolate the impact of Triton on strip development and signal generation, we compared conditions with and without 0.1% Triton X-100 by monitoring control line development in the absence of N protein. This design intentionally maximizes the amount of AuNS-DNA-Net conjugate available to reach the control line in both cases, thereby avoiding conjugate depletion at the test line and eliminating capture competition effects. Under these no antigen conditions, we quantified the capillary wetting front progression on the nitrocellulose membrane (NCM) as a direct readout of migration behavior. The wetting front reached the control line position in approximately 72 s with 0.1% Triton X-100 and approximately 110 s without Triton, indicating faster wicking in the presence of the Triton surfactant. Using the Lucas Washburn relation for porous substrates, $x^2 = k*t$, where x denotes the NCM length traversed by the wetting front and t denotes the corresponding wicking time, we used x = 2 cm to estimate the apparent wicking coefficients[1]. This resulted in k = 0.0556 cm^2 s^{-1} with Triton and k = 0.0364 cm^2 s^{-1} without Triton conditions, corresponding to an approximately 1.5-fold increase in the wicking coefficient. Prior studies similarly report that Triton X-100 can enhance wettability by lowering interfacial resistance and accelerating liquid transport in porous materials[4]. Importantly, this faster wetting did not compromise practical strip development or endpoint signal formation. In the same no antigen condition, the control line developed robustly in both cases within a fix acquisition window, with control line intensities of 0.71 for 0.1% Triton X-100 **(Movie S2)** and 0.65 without Triton **(Movie S3)**, indicating that inclusion of Triton is compatible with normal migration and assay operation under our conditions.

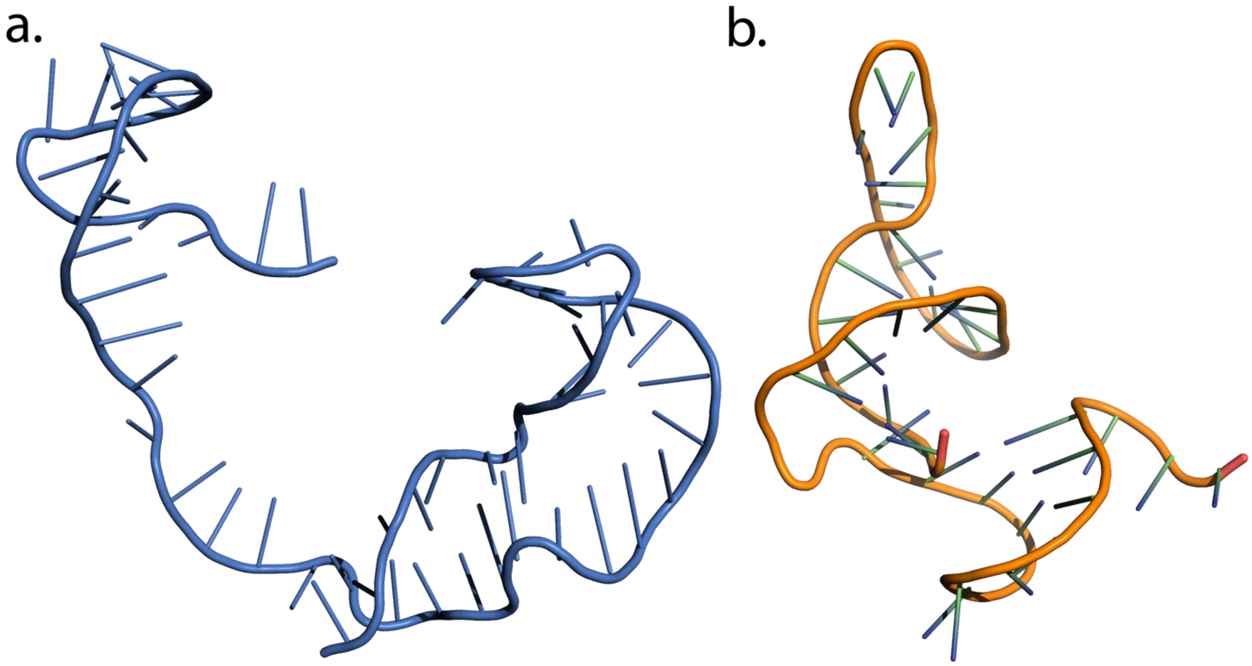

Figure S1. Predicted secondary structures of DNA aptamers. (a) Apt1 (PEA1-3) and **(b)** Apt2 (PEA2). Structures were generated using the 3dDNA webserver[5], which constructs three-dimensional DNA models based on input sequences and secondary structures predicted by RNAfold.

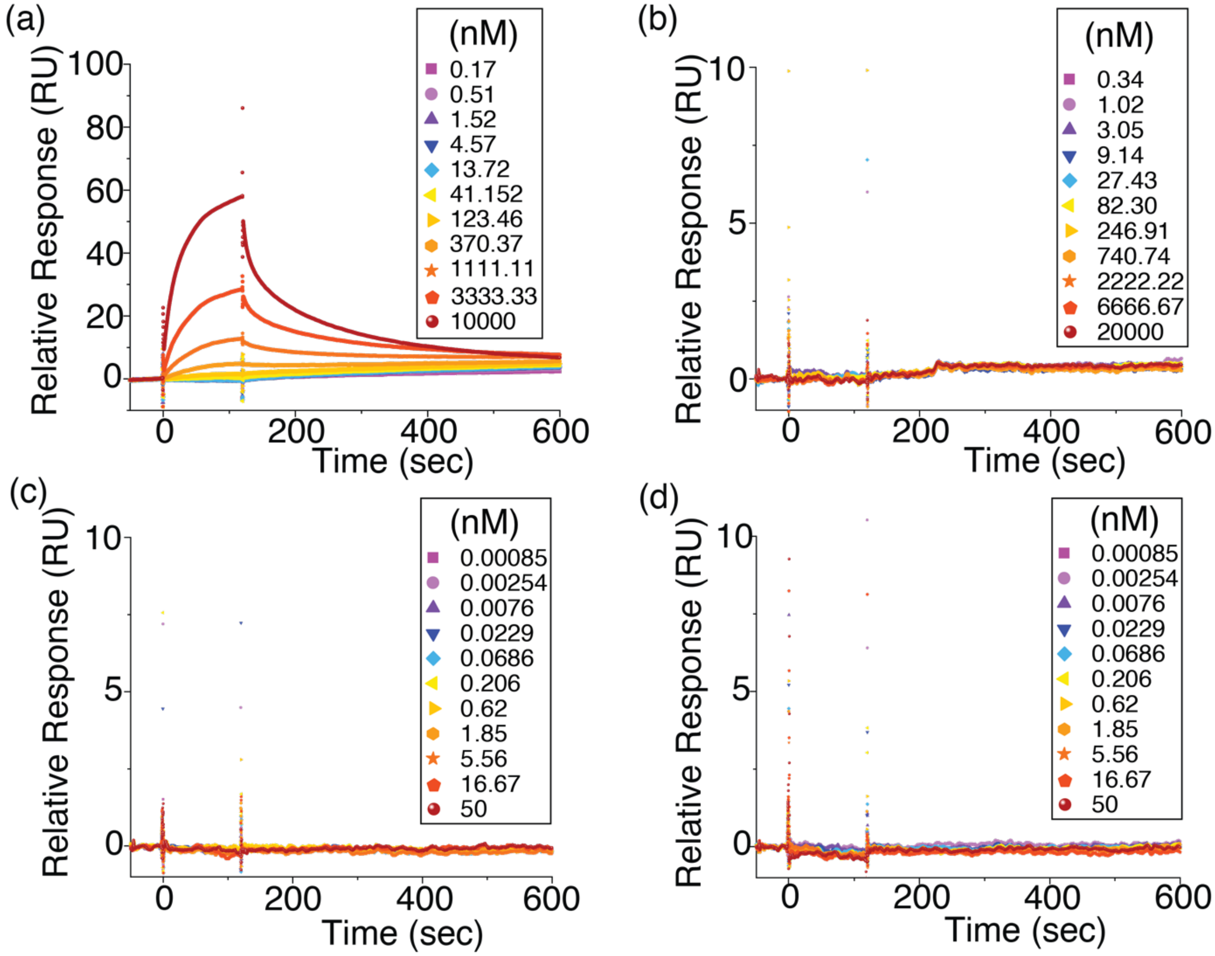

Figure S2. Binding affinities of positive and negative controls assessed by surface plasmon resonance (SPR). (a) A PEDV-N protein-specific monoclonal antibody was used as a positive control, yielding a dissociation constant (K_D) of 60.32 pM. Negative controls included: **(b)** A non-target influenza aptamer[2]. **(c)** a DNA-Net construct without aptamer functionalization, and **(d)** a DNA-Net functionalized with the influenza-specific aptamer were all tested against the PEDV-N protein and showed no significant binding. All experiments were performed in triplicate, with similar results across replicates.

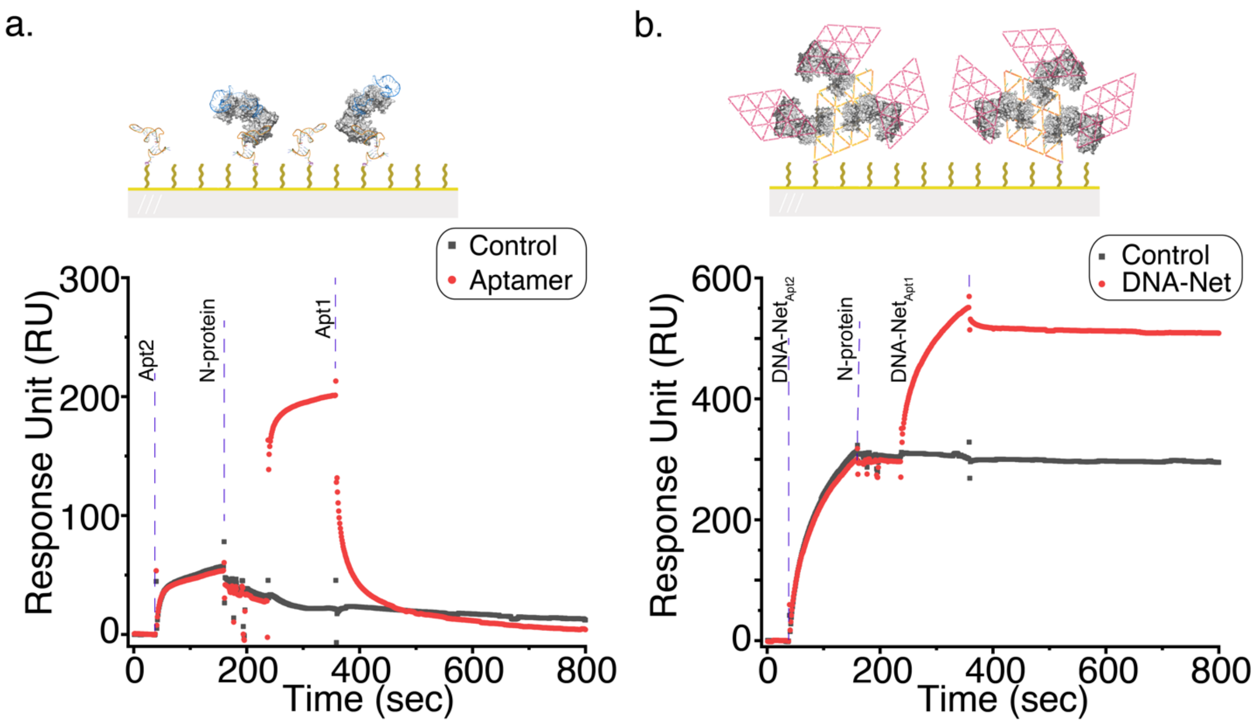

Figure S3. SPR validation of sandwich complex formation using free aptamers and DNA Net integrated aptamers. (a) Sequential injection SPR sensorgrams assessing sandwich formation with free aptamers. Biotinylated Apt2 was captured on a streptavidin sensor surface, followed by serial injection of recombinant PEDV N protein and then Apt1. The increase in response upon N protein injection confirms binding to surface immobilized Apt2. After a brief 30 s running buffer wash to remove unbound N protein, injection of Apt1 produced an additional increase in response (red traces), consistent with formation of an Apt2–N protein–Apt1 sandwich complex. A non-target influenza aptamer injected in place of Apt1 (gray traces) yielded minimal additional response, supporting aptamer specific secondary binding rather than nonspecific adsorption. **(b)** Sequential injection SPR sensorgrams assessing sandwich formation with DNA-Net displayed aptamers. Biotinylated DNA-Net$_{Apt2}$ was captured on a streptavidin sensor surface, followed by serial injection of recombinant PEDV N protein and then DNA-Net$_{Apt1}$. The additional response increase during DNA-Net$_{Apt1}$ injection indicates that DNA-Net presentation preserves non overlapping aptamer recognition and supports the DNA-Net$_{Apt2}$–N protein–DNA-Net$_{Apt1}$ sandwich configuration. A control DNA-Net bearing non-target influenza aptamer showed minimal response throughout the sequence, indicating that binding is not driven by nonspecific interactions with the DNA-Net scaffold. Schematics above each panel illustrate the corresponding binding configurations. Responses are plotted in resonance units as a function of time. Experiments were repeated with n = 3 biologically independent replicates.

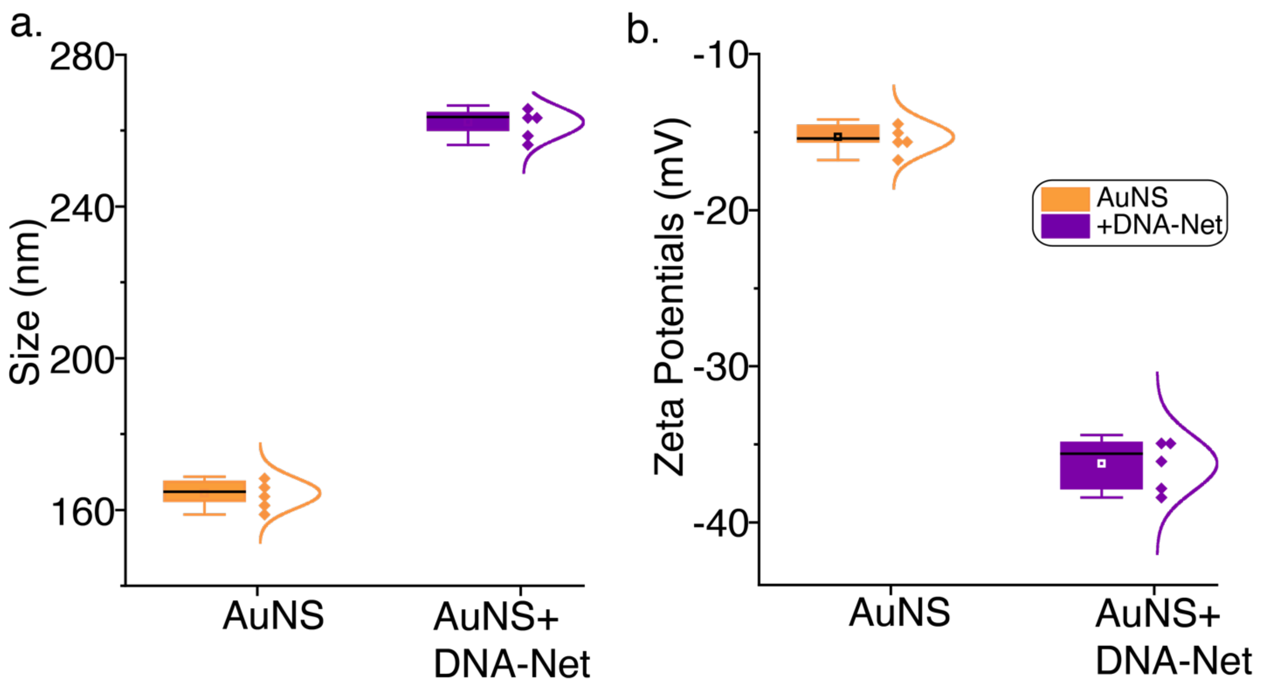

Figure S4. Conjugation of DNA-Net with gold nanoshells (AuNS). (a) Dynamic light scattering (DLS) analysis shows an increase in hydrodynamic diameter for AuNS conjugated with DNA-Net compared to unmodified AuNS. **(b)** Zeta potential measurements indicate a decrease upon DNA-Net conjugation, suggesting improved colloidal stability. Data are presented as mean ± SD, n = 5 biologically independent samples.

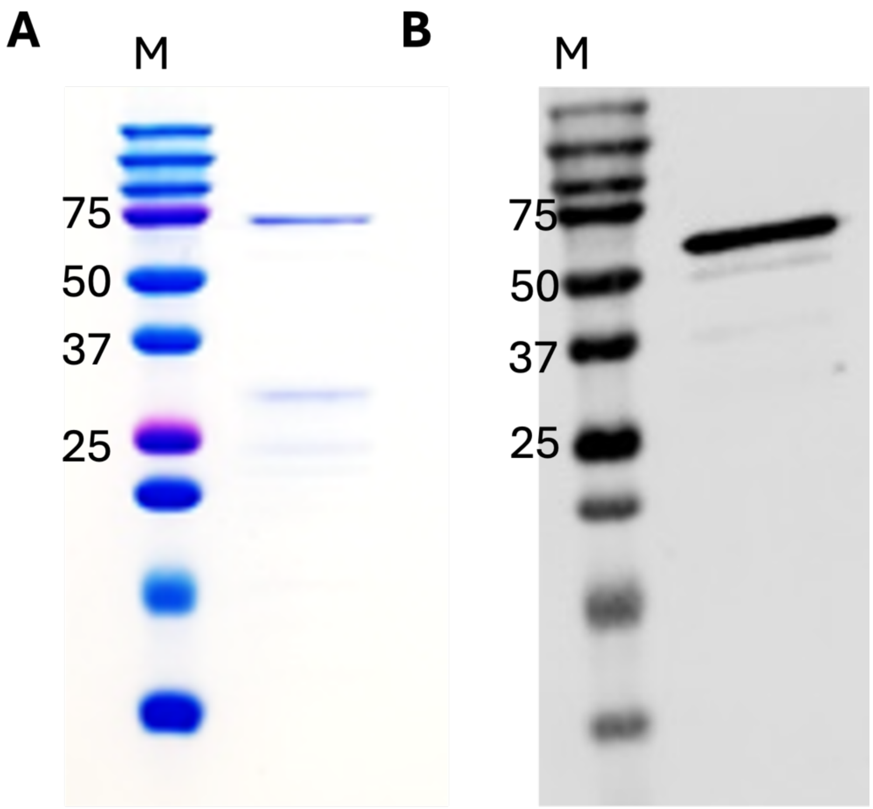

Figure S5. PEDV-N protein expression and characterization. **(A)** SDS-PAGE analysis of purified recombinant PEDV-N protein visualized by Coomassie Brilliant Blue staining. **(B)** Western blot to detect PEDV-N protein using a mouse monoclonal Antibody.

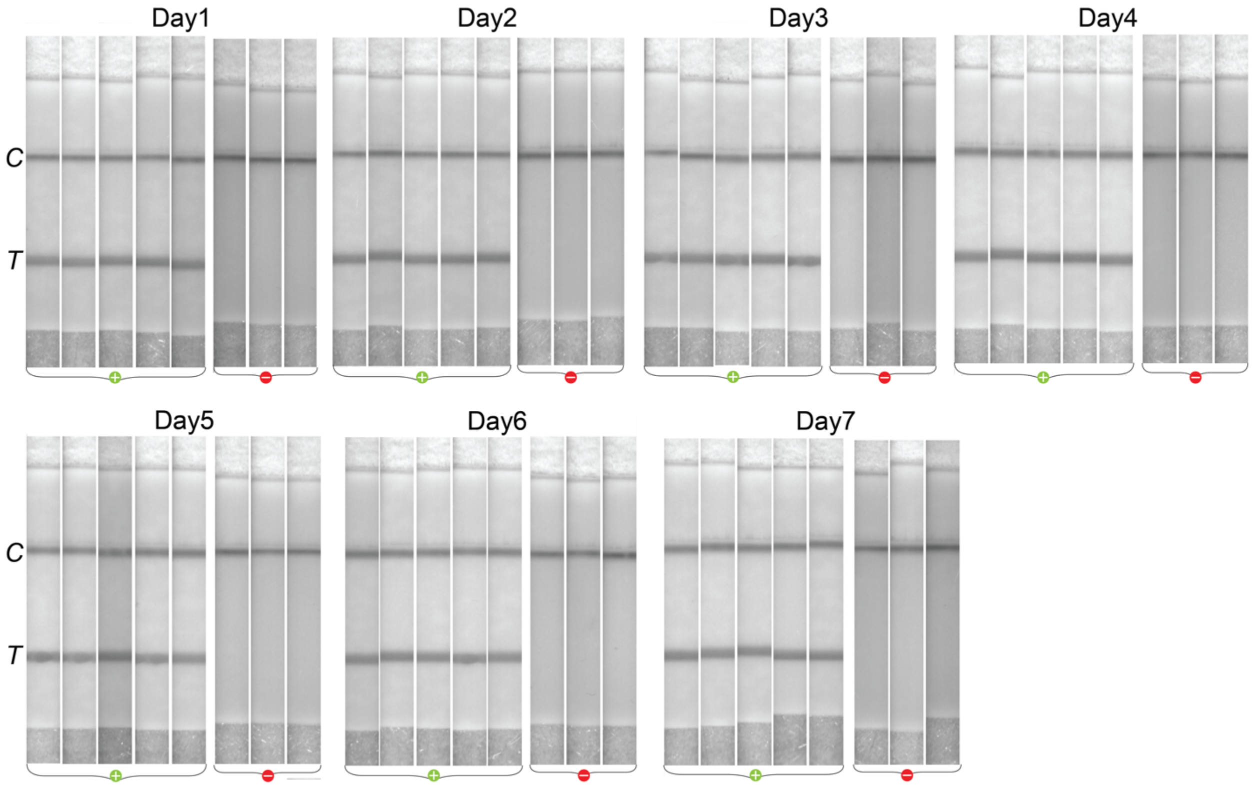

Figure S6. Repeatability of the lateral flow assay was evaluated over seven consecutive days. Representative test strip images acquired on Day 1 through Day 7 are shown for positive and negative control conditions. The plus symbol indicates 108 ng per mL N protein spiked into 30% porcine oral fluid, whereas the minus symbol indicates no protein added. On each day, replicate strips were run using identical assay workflow and readout conditions to assess day to day consistency of signal development. C and T denote the control and test lines. Data represent mean ± SD from n=5 biologically independent replicates.

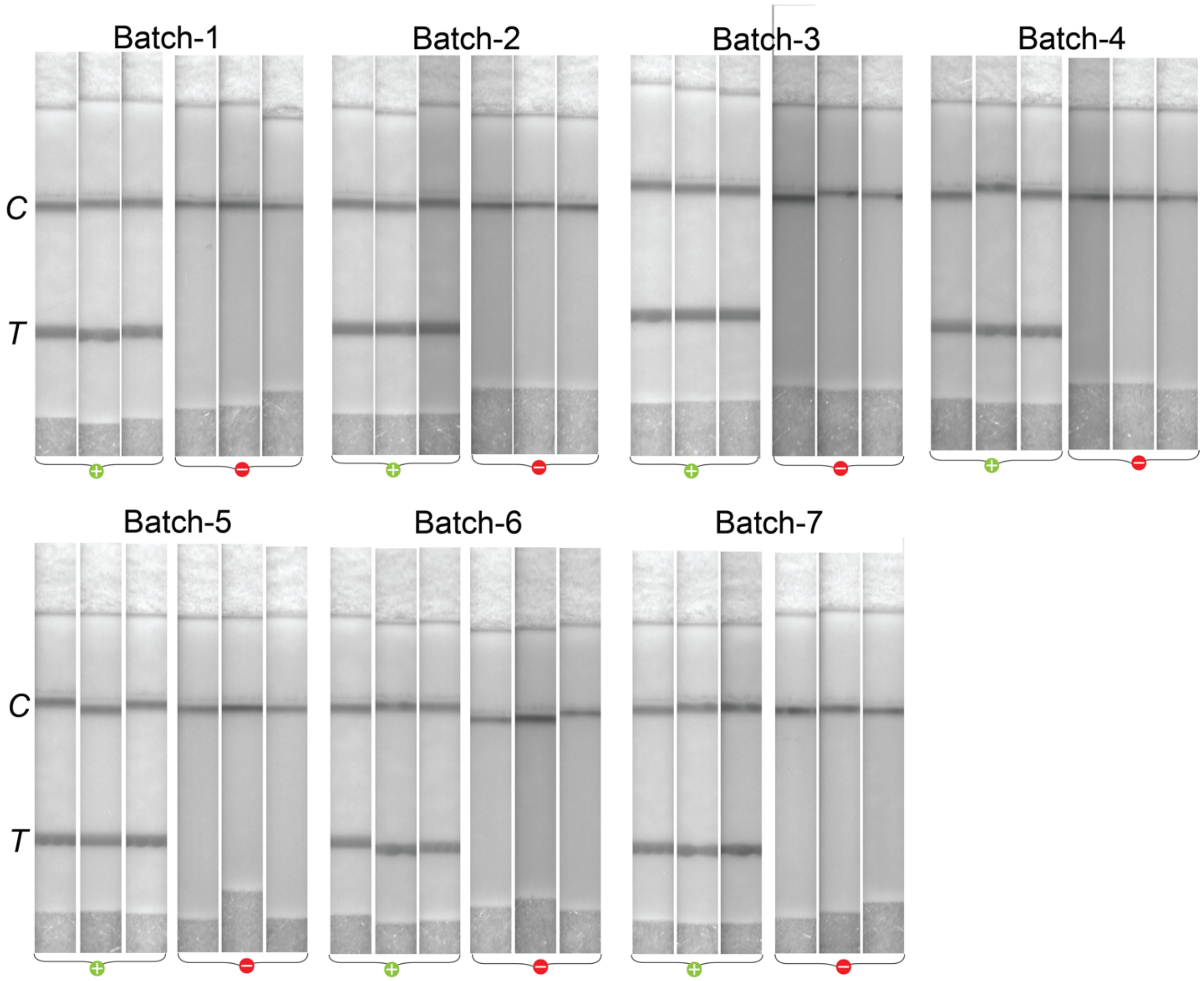

Figure S7. Reproducibility of the lateral flow assay across independently prepared AuNS-DNA Net conjugate batches. Representative test strip images obtained using seven distinct conjugate batches (Batches 1 to 7) that were synthesized on different days and independently integrated into the LFA workflow to evaluate batch to batch consistency of signal generation. The plus symbol denotes 108 ng per mL N protein spiked in 30% porcine oral fluid, while the minus symbol denotes no protein added. For each batch, replicate strips were run under identical assay and readout conditions, and signals were quantified to assess reproducibility. C and T denote the control and test lines. Data represent mean ± SD from n=3 biologically independent replicates.

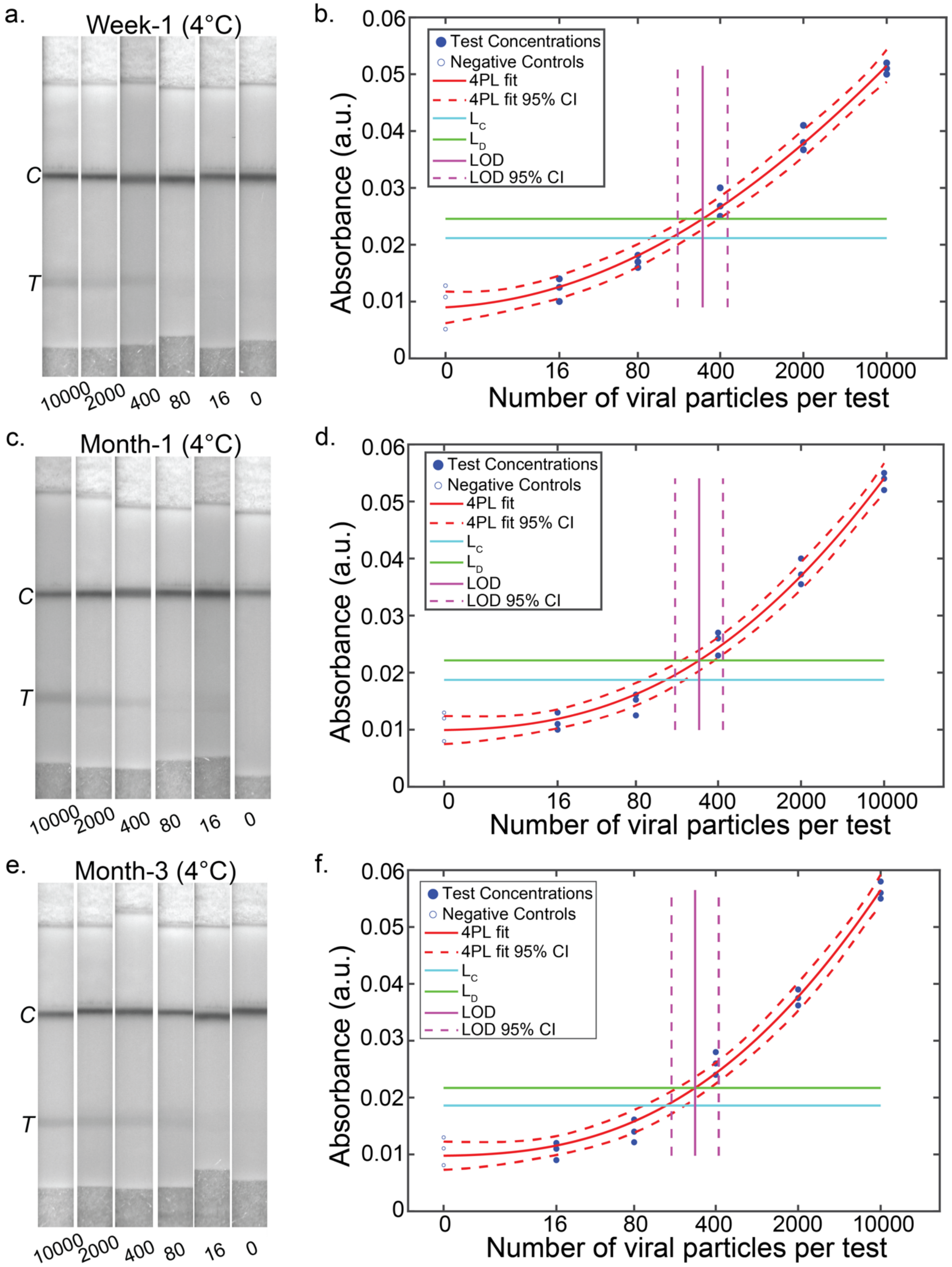

Figure S8. Analytical performance and limit of detection of the lateral flow assay following storage at 4°C. Representative test strip images after storage for 1 week **(a)**, 1 month **(c)**, and 3

months **(e)**, respectively, measured across the indicated viral particles per test (10,000 to 16). Wild type PEDV virions were lysed with 0.1% Triton X-100 and serially diluted in 1X PBS containing 30% porcine oral fluid. A matrix matched buffer composed of 1X PBS with 30% porcine oral fluid served as the negative control (0 virus). C and T denote the control and test lines. **(b,d,f)** Corresponding curves showing test line absorbance as a function of viral particles per test, fit using a four-parameter logistic (4PL) model. Filled circles represent test concentrations and open circles represent negative controls. The solid red line shows the 4PL fit and dashed red lines indicate the 95% confidence interval (CI). Horizontal lines indicate the limit of blank L_C and the signal domain detection threshold L_D. The vertical magenta line denotes the calculated LOD and dashed magenta lines indicate the 95% CI for the LOD. The measured LOD values were 284.51 viral particles per test (1 week, **subpanel b**), 276.75 viral particles per test (1 month, **subpanel d**), and 266.73 viral particles per test (3 months, **subpanel f**). Data represent mean ± SD from n=3 biologically independent replicates.

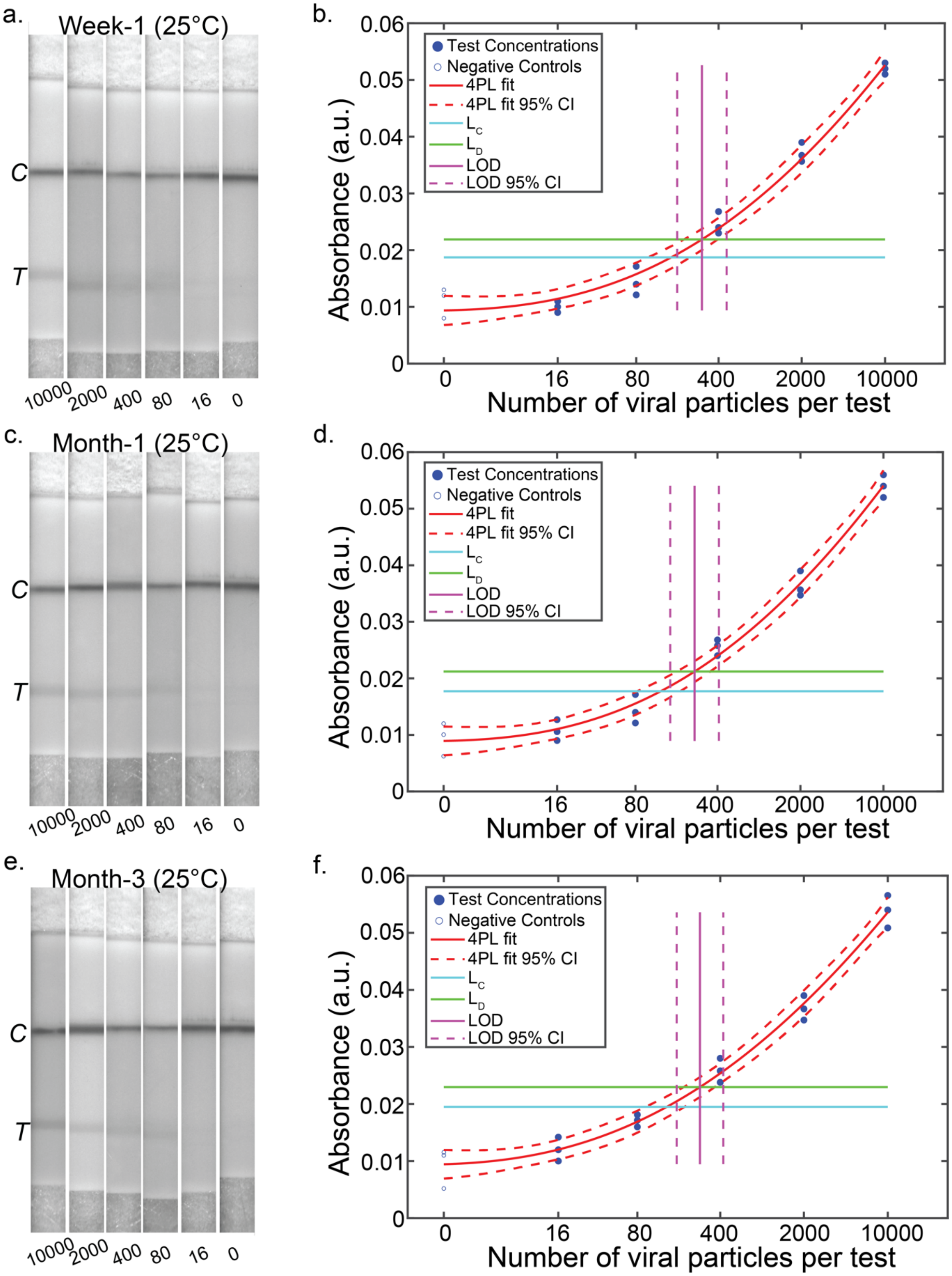

Figure S9. Lateral flow assay performance and limit of detection following storage at 25° C. Representative test strips obtained after 1 week **(a)**, 1 month **(c)**, and 3 months **(e)** of storage,

respectively, tested across the indicated viral particle loads per assay (10,000 to 16). Wild type PEDV virions were lysed with 0.1% Triton X 100 and serially diluted in 1X PBS supplemented with 30% porcine oral fluid. A matrix matched negative control consisting of 1X PBS with 30% porcine oral fluid was included (0 virus). **(b,d,f)** Corresponding dose response curves of test line absorbance versus viral particles per test, modeled using a four-parameter logistic (4PL) fit. The solid red line indicates the 4PL fit and dashed red lines represent the 95% confidence band. Horizontal lines mark the limit of blank L_C and the signal domain detection threshold L_D. The vertical magenta line indicates the calculated LOD and dashed magenta lines indicate the 95% confidence interval. The measured LOD values were 289.44 viral particles per test (1 week, **subpanel b**), 256.03 viral particles per test (1 month, **subpanel d**), and 270.27 viral particles per test (3 months, **subpanel f**). Data represent mean ± SD from three biologically independent replicates.

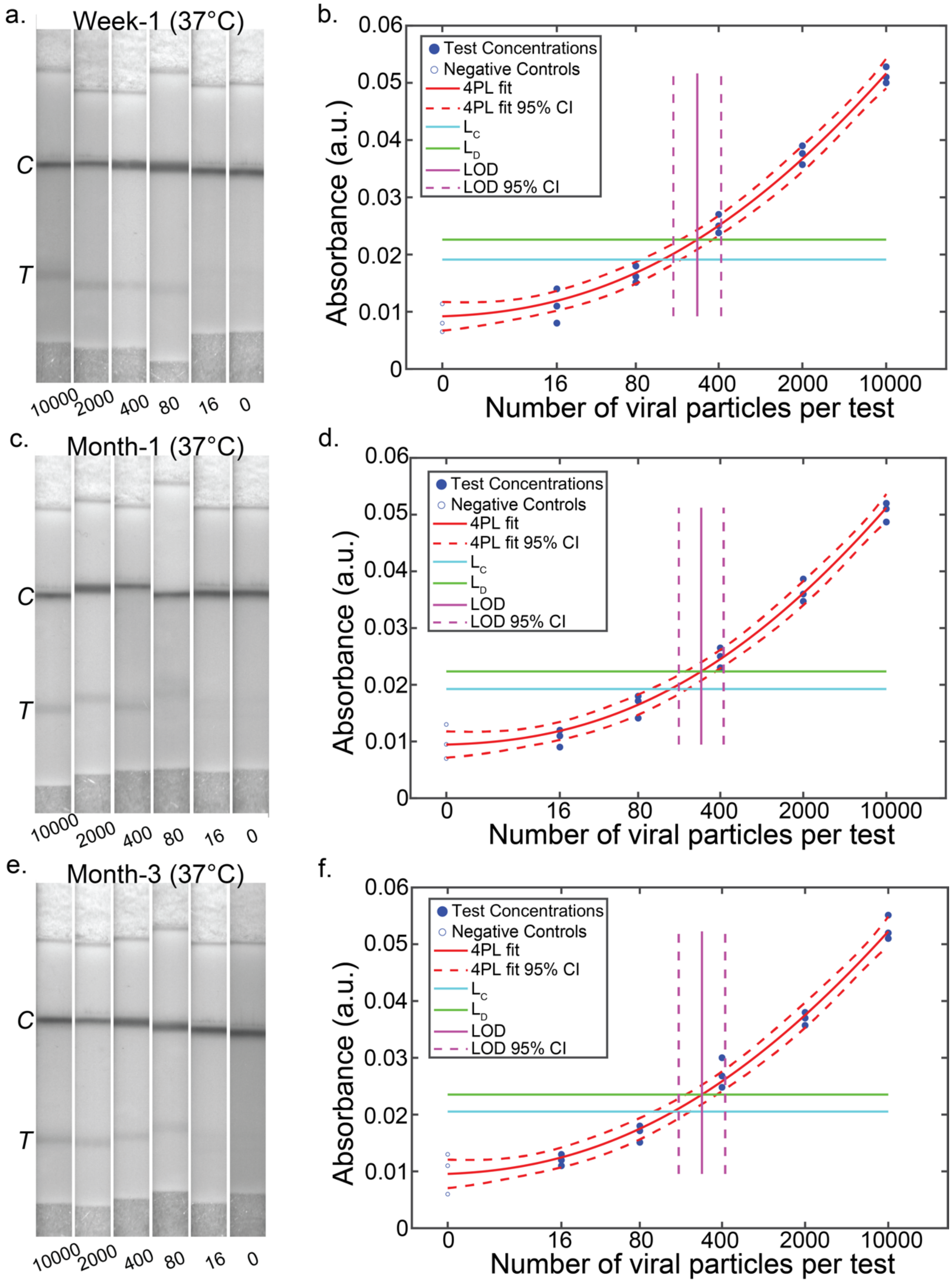

Figure S10. Lateral flow assay analytical performance and limit of detection after storage at 37° C. Representative test strips collected after 1 week **(a)**, 1 month **(c)**, and 3 months **(e)** of storage, respectively, evaluated across the indicated viral particle inputs per test (10,000 to 16).

Wild type PEDV virions were lysed with 0.1% Triton X 100 and serially diluted in 1× PBS containing 30% porcine oral fluid. A matrix matched negative control of 1× PBS with 30% porcine oral fluid was included (0 virus). **(b,d,f)** Corresponding calibration curves of test line absorbance versus viral particles per test, fit using a four-parameter logistic (4PL) model. The solid red line denotes the 4PL fit and dashed red lines indicate the 95% confidence band. Horizontal lines denote the limit of blank L_C and the signal domain detection threshold L_D. The vertical magenta line marks the calculated LOD and dashed magenta lines indicate the 95% confidence interval. The measured LOD values were 264.55 viral particles per test (1 week, **subpanel b**), 275.85 viral particles per test (1 month, **subpanel d**), and 270.56 viral particles per test (3 months, **subpanel f**). Data represent mean ± SD from three biologically independent replicates.

Table S1. Cross-platform comparison of DNA nanostructure-enabled lateral flow assays, highlighting the advantages of a dual-aptamer DNA-Net sandwich LFA.

Dimension / aspect	Prior DNA-Net LFA (SARS-CoV-2)	DNA multiway-junction LFA platform	Existing PEDV diagnostics (antibody LFAs, electrochemical aptasensor)	This work – PEDV DNA-Net LFA (highlighted features)
**Core principle**	Multivalent DNA-Net displaying trimeric clusters of a single spike aptamer to enhance binding to SARS CoV 2 virions	Target-induced 3-/4-way DNA junctions bridge AuNPs and capture strands on a generic strip	Antibody–antigen binding on colloidal gold LFAs; single aptamer on electrochemical sensor	Multivalent DNA-Net carrying two non-competing PEDV N aptamers arranged in trivalent clusters and split between reporter and capture Nets to form a dual aptamer sandwich for N protein
**Target type**	Surface glycoprotein on intact human SARS-CoV-2 virions	Purified molecular targets (nucleic acids, proteins, small molecules, enzymes)	PEDV nucleocapsid protein	PEDV nucleocapsid protein released by controlled Triton X 100 lysis of intact virus in pig oral fluid, a conserved internal antigen chosen to improve coverage across PEDV variants
**Sample matrix and pretreatment**	Diluted human saliva, serum, and urine, no detergent lysis	Buffer, diluted serum, cell lysate, with target specific preparation and often pre-amplification for nucleic acids	Swine feces, intestinal contents, or oral fluid; usually processed in laboratory or basic field setting; electrochemical format requires electrode handling and instrumentation	Swine oral fluid collected on farm, subjected to 0.1% Triton X-100 lysis for lateral flow and minimal matrix interference; directly applied to strip without amplification or purification

**Mechanistic architecture**	Geometry tuned pattern matching of spike trimers using one spike aptamer type on DNA Net, no sandwich requirement and no detergent	Multibranch DNA junction acts as a generic bridging nanostructure between AuNP and test line, not geometrically tuned to a specific viral antigen	Antibodies or a single aptamer provide monovalent or effectively low valency binding on planar surfaces or in solution	Dual aptamer DNA Net implementation where two epitope distinct N aptamers are co displayed in each trivalent cluster and distributed on reporter and capture Nets to maintain a true sandwich around N protein in solution after lysis
**Assay format and instrumentation**	Colorimetric lateral flow test for human COVID use, read visually or with simple scanner	Colorimetric lateral flow test, designed as a reconfigurable platform for many molecular targets	Antibody LFAs are colorimetric and reader free but often less sensitive; electrochemical aptasensor requires potentiostat and at least one hour assay time	Colorimetric lateral flow test tailored to veterinary on farm use, fully synthetic DNA components, no reader or instrument required and ten-minute total assay time
**Need for amplification or complex sample processing**	No nucleic acid amplification, samples are relatively clean bodily fluids	Often requires nucleic acid amplification for DNA targets such as RPA for HBV DNA although readout is still on strip	qRT PCR and isothermal assays require extraction and thermocycling; electrochemical aptasensor requires laboratory processing; antibody LFAs need no amplification but have higher LoD	No nucleic acid amplification and no extraction step while still reaching near PCR level LOD in oral fluid owing to multivalent DNA Net mediated enhancement of N binding

**Analytical sensitivity**	LoD in low 10^3–10^4 copies mL^{-1} for SARS-CoV-2 variants	LoDs in nM–pM or low-U μL^{-1} range, target dependent, mainly for purified or mildly complex samples	antibody LFAs typically ≈10^4–10^5 $TCID_{50}$ mL^{-1}; Electrochemical aptasensor LoD in sub-µg mL^{-1} for PEDV N	LOD ≈ 1.33 ng mL^{-1} PEDV N in oral fluid, ≈ 278 viral copies per test, substantially lower than previous LFAs and PEDV aptasensor
**Clinical validation context**	Human respiratory infection; compared mainly to commercial COVID antigen LFAs	Demonstration on selected clinical / pseudo-clinical samples, not focused on farm virology	Used for PEDV surveillance and outbreak confirmation, largely in diagnostic laboratories or with limited pen side sensitivity and reliance on instruments for molecular methods	Pig oral fluid samples evaluated against qRT PCR, sensitivity ~95% and specificity ~97%, including detection up to relatively high Ct values relevant for early and subclinical infection
**Translational focus**	Human self-testing and POC COVID-19 screening	Versatile molecular sensing platform for many molecular targets	Routine PEDV surveillance and outbreak confirmation, mostly lab-based; limited pen-side sensitivity	Field-deployable PEDV surveillance tool that closes much of the gap between simple antigen strips and qRT-PCR for non-invasive oral-fluid testing in barns
**What is shared vs. new**	Shares conc ept of multivalent DNA-Net for binding enhanceme nt	Shares concept of DNA-based multivalent bridging in LFAs	Shares use of aptamers / antibodies for PEDV	New combination of: dual-aptamer DNA-Net architecture tailored to PEDV N, detergent-lysed pig oral fluid as matrix, and on-farm clinical validation with near-PCR-level sensitivity in a reader-free LFA format

Table S2. Non-covalent interactions identified between DNA aptamer1 (Apt1) and the PEDV-N protein.

Apt1 (PEA1-3) interaction with PEDV-N protein				
Aptamer	Bases	N-protein (Index)	Amino Acids	Type of interactions
A	1	325	T	H-Bond
A	1	473	E	H-Bond
A	1	474	W	H-Bond
A	1	476	R	H-Bond
A	1	486	C	H-Bond
G	2	470	D	H-Bond
C	3	330	D	H-Bond
T	7	16	E	H-Bond
A	9	14	K	H-Bond
A	9	142	E	H-Bond
C	11	481	N	H-Bond
T	19	289	A	Hydrophobic
T	19	290	Q	Hydrophobic
T	20	287	G	H-Bond
G	21	338	S	H-Bond
C	22	338	S	H-Bond
T	23	338	S	H-Bond
T	24	344	K	H-Bond
C	38	633	N	H-Bond
T	42	666	D	H-Bond
T	42	668	N	H-Bond
G	47	802	R	H-Bond
C	49	807	E	H-Bond
T	50	796	K	Salt Bridge

Table S3. Analysis of non-covalent binding interactions between PEDV-N protein and DNA aptamer2 (Apt2).

Apt2 (PEA2) interaction with PEDV-N protein				
Aptamer	Bases	N-protein (Index)	Amino Acids	Type of interactions
C	1	21	A	H-Bond
C	2	17	N	H-Bond
A	3	16	E	H-Bond
C	9	470	d	H-Bond
T	11	861	E	H-Bond
T	11	470	D	H-Bond
T	11	476	R	H-Bond
C	20	16	E	H-Bond
C	20	40	K	Salt Bridge
T	21	16	E	H-Bond
T	21	40	K	Salt Bridge
G	23	24	G	H-Bond
T	26	16	E	H-Bond
C	28	2	D	H-Bond
C	32	73	D	H-Bond
C	32	434	P	Hydrophobic
C	32	4	K	Salt Bridge
A	34	5	D	H-Bond
A	34	10	R	Salt Bridge
G	35	1	R	H-Bond
G	35	2	D	H-Bond
G	36	160	D	H-Bond
G	36	222	S	H-Bond
G	36	223	Q	H-Bond
G	36	2	D	H-Bond
G	37	161	A	H-Bond
G	37	228	K	H-Bond
T	39	228	K	H-Bond
T	40	225	D	H-Bond

Table S4. SPR kinetic parameters and equilibrium dissociation constants for Apt1, Apt2, DNA Net_{Apt1}, DNA Net_{Apt2}, and mAb binding to PEDV N protein, obtained by 1:1 Langmuir fitting in BIAevaluation (K_D = kd/ka).

Parameters	Apt1	Apt2	DNA-Net_{Apt1}	DNA-Net_{Apt2}	mAb
Association rate constant-ka, (1/Ms)	$3.243*10^9$	$6.11*10^6$	$1.682*10^7$	$1.53*10^7$	$4.32*10^5$
Dissociation rate constant- kd (1/s)	0.3384	$1.86*10^{-4}$	$2.083*10^{-6}$	$1.21*10^{-6}$	$2.61*10^{-5}$

Table S5. Matrix dependent limit of detection calculations for buffer, oral fluid, and WT virus samples

**Description**	**Value**	**Buffer**	**Oral Fluid**	**WT Virus**
		**(Figure 3b)**	**(Figure 4b)**	**(Figure 4d)**
**Limit of Blank**	L_C	0.013919986	0.016	0.018127018
**Limit of Detection in Signal Domain**	L_D	0.024416032	0.02426798	0.023720664
**Curve Fit between Signal and Concentration Domains**	4-parameter logistic	a = 0.0012 (-0.014, 0.017)	a = 0.013 (0.0087, 0.017)	a = 0.017 (0.01387, 0.020)
		b = 1.373 (0.83, 1.91)	b = 2.362 (1.92, 2.81)	b = 3.571 (1.383, 5.76)
		c = 20.47 (-120.9, 161.9)	c = 2.064 (1.69, 2.43)	c = 8.237 (-13.02, 29.5)
		d = 5.351 (-37.26, 47.96)	d = 0.3156 (0.2548, 0.3765)	d = 0.5377 (-3.17, 4.25)
**Limit of Detection (ng/mL)**	LOD with 95% CI	0.4602 [0.023, 0.99]	1.3276 [0.67, 2.14]	274.2419 [97.99, 761.16]

Table S6. Day to day repeatability metrics for the lateral flow assay across seven consecutive days

**Repeatability Testing**							
**Parameters**	**Day1**	**Day2**	**Day3**	**Day4**	**Day5**	**Day6**	**Day7**
**Mean**	0.154	0.157	0.153	0.157	0.153	0.159	0.157
**SD**	0.003	0.004	0.002	0.003	0.004	0.004	0.004
**Intra-assay CV (%)**	1.975	2.694	1.570	1.947	2.357	2.244	2.467
**Inter-assay CV (%)**	2.179						

Table S7. Batch to batch reproducibility metrics for the lateral flow assay across seven independently prepared AuNS–DNA Net conjugate batches

Reproducibility Testing							
**Parameters**	**Day1**	**Day2**	**Day3**	**Day4**	**Day5**	**Day6**	**Day7**
**Mean**	0.153	0.155	0.154	0.155	0.156	0.157	0.159
**SD**	0.003	0.005	0.003	0.003	0.004	0.004	0.004
**Intra-assay CV (%)**	2.038	3.482	2.077	2.007	2.460	2.835	2.546
**Inter-assay CV (%)**	2.492						

Table S8. Statistical limit of detection analysis for the 4°C stability study across different storage time points (1 week, 1 month, and 3 months)

**Description**	**Value**	**4^0C (SI Figure X)**		
		**1 Week**	**1 Month**	**3 Month**
**Limit of Blank**	L_C	0.0212	0.0187	0.0186
**Limit of Detection in Signal Domain**	L_D	0.0246	0.0221	0.0217
**Curve Fit between Signal and Concentration Domains**	4-parameter logistic	a = 0.008 (0.0057, 0.012)	a = 0.0099 (0.0074, 0.0125)	a = 0.0097 (0.0072, 0.0123)
		b = 2.167 (0.7201, 3.613)	b = 2.748 (1.116, 4.38)	b = 2.883 (1.231, 4.535)
		c = 11.77 (-56.77, 80.32)	c = 9.243 (-28.09, 46.58)	c = 8.801 (-23.24, 40.84)
		d = 0.4941 (-4.452, 5.44)	d = 0.4951 (-3.842, 4.832)	d = 0.5106 (-3.707, 4.729)
**Limit of Detection (ng/mL)**	LOD with 95% CI	284.51 [174.67, 462.65]	276.75 [173.45, 440.85]	266.73 [167.58, 423.85]

Table S9. Limit of detection analysis for the 25°C stability data set across storage durations (1 week, 1 month, and 3 months)

Description	Value	25^{0}C (SI Figure X)		
		**1 Week**	**1 Month**	**3 Month**
**Limit of Blank**	L_C	0.018	0.0177	0.0195
**Limit of Detection in Signal Domain**	L_D	0.022	0.0212	0.023
**Curve Fit between Signal and Concentration Domains**	4-parameter logistic	a = 0.0093 (0.00665, 0.012)	a = 0.00889 (0.0062, 0.01154)	a = 0.0094 (0.0067, 0.0120)
		b = 2.686 (1.003, 4.37)	b = 2.706 (1.096, 4.317)	b = 2.497 (1.027, 3.966)
		c = 9.689 (-35.2, 54.58)	c = 9.367 (-29.1, 47.83)	c = 10.54 (-40.23, 61.3)
		d = 0.5186 (-4.637, 5.674)	d = 0.5059 (-3.943, 4.955)	d = 0.5498 (-4.767, 5.866)
**Limit of Detection (ng/mL)**	LOD with 95% CI	289.44 [178.71, 467.99]	256.03 [158.87, 411.81]	270.27 [172.08, 423.83]

Table S10. Limit of detection calculations for the 37° C stability condition across storage times (1 week, 1 month, and 3 months)

Description	Value	37^{0}C (SI Figure X)		
		**1 Week**	**1 Month**	**3 Month**
**Limit of Blank**	L_C	0.0191	0.0193	0.0205
**Limit of Detection in Signal Domain**	L_D	0.0226	0.0223	0.0235
**Curve Fit between Signal and Concentration Domains**	4-parameter logistic	a = 0.0091 (0.0064, 0.012)	a = 0.0094 (0.0069, 0.012)	a = 0.0095 (0.0067, 0.012)
		b = 2.424 (0.9561, 3.891)	b = 2.521 (1.082, 3.96)	b = 2.364 (0.9434, 3.784)
		c = 10.51 (-39.66, 60.68)	c = 9.735 (-29.19, 48.66)	c = 10.93 (-43.64, 65.5)
		d = 0.4935 (-4.041, 5.028)	d = 0.4449 (-3.058, 3.948)	d = 0.5128 (-4.315, 5.341)
**Limit of Detection (ng/mL)**	LOD with 95% CI	264.55 [166.46, 419.75]	275.85 [178.11, 426.64]	270.56 [171.88, 425.23]

Table S11. Summary of clinical samples and metadata for LFA device evaluation

Sample ID	PEDV Ct	Source/Sample type	Farm Type and Location,	Pig age	Notes
1	14.03	Feces	Not available	Not Available	2025
2	14.03	Fecal Swab	North Fork, IL	Not Available	-
3	14.7	Fecal Swab	Iowa, Nursery	3 Weeks	PEDV +, PDCoV +
4	15	Fecal Swab	Oklahoma, Sow	2 Years	-
5	16.33	Feces	Bradford, IL	Not Available	12/22/21
6	17	Fecal Swab	Oklahoma, Sow	2 Years	-
7	17.6	Fecal Swab	Iowa, Grow-Finish	11 Weeks	PEDV +, PDCoV +
8	17.8	Intestine	Rock City, IL	Not Available	2/10/22
9	18.1	Fecal Swab	Iowa, Nursery	5 Weeks	-
10	18.6	Oral Fluid	Iowa, Grow-Finish	6 Months	PEDV +, PDCoV +
11	18.6	Oral Fluid	Oklahoma, Grow-Finish	10 Weeks	PEDV +, PDCoV -
12	18.71	Fecal Swab	North Fork, IL	Not Available	PEDV
13	19.1	Fecal Swab	North Fork, IL	Not Available	PEDV
14	19.3	Fecal Swab	Iowa	Not Available	PEDV +, PDCoV +
15	19.3	Oral Fluid	Oklahoma, Grow-Finish	10 Weeks	-
16	20.07	Culture Material	Forrest, IL	Not Available	3/9/21
17	20.1	Oral Fluid	Oklahoma, Grow-Finish	Adult	PEDV +, PDCoV -
18	20.11	Intestine and Stomach Contents	Not available	Not Available	PEDV
19	20.28	Feces	Willow Hill, IL	Not Available	3/31/23
20	20.32	Feces	Not available	Not Available	3/7/22
21	20.32	Feces	Bradford, IL	Not Available	12/22/21
22	20.4	Oral Fluid	Oklahoma, Grow-Finish	13 Weeks	PEDV +, PDCoV -
23	20.55	Feces	Raymond, IL	Not Available	3/7/22
24	21.2	Oral Fluid	Oklahoma, Grow-Finish	13 Weeks	PEDV +, PDCoV -
25	21.3	Fecal Swab	Iowa	Not Available	PEDV +, PDCoV +
26	21.5	Oral Fluid	Oklahoma, Grow-Finish	21 Weeks	PEDV +, PDCoV -

27	21.89	Intestine Pool A&B in RLT	Lexington, IL	1 Week	PEDV
28	22.4	Oral Fluid	Oklahoma, Grow-Finish	13 Weeks	PEDV +, PDCoV -
29	22.64	Feces	Stewardson, IL	Not Available	5/19/22
30	23.19	Feces	Raymond, IL	Not Available	5/9/22
31	23.26	Feces	Raymond, IL	Not Available	3/7/22
32	23.33	Feces	Sigel, IL 62462	Not Available	6/10/21
33	24.2	Feces	Raymond, IL	Not Available	3/7/22
34	24.38	Feces	Not available	Not Available	4/25/22
35	25	Oral Fluid	Oklahoma, Grow-Finish	21 Weeks	PEDV +, PDCoV -
36	25.2	Fecal Swab	Oklahoma, Sow	2 Years	PEDV +, PDCoV -
37	25.6	Oral Fluid	Oklahoma, Grow-Finish	13 Weeks	PEDV +, PDCoV -
38	25.67	Feces	Rock City, IL	Not Available	2/10/22
39	26.09	Feces	Bradford, IL	Not Available	12/22/21
40	26.23	Feces	Raymond, IL	Not Available	5/9/22
41	26.37	Feces	Raymond, IL	Not Available	5/9/22
42	27.08	Feces	Not available	Not Available	4/25/22
43	27.84	Feces	Bradford, IL/61421	Not Available	1/11/22
44	27.5	Fecal Swab	Oklahoma, Grow-Finish	18 Weeks	PEDV +, PDCoV -
45	28	Oral Fluid	Oklahoma, Grow-Finish	18 Weeks	PEDV +, PDCoV -
46	28.1	Oral Fluid	Oklahoma, Grow-Finish	10 Weeks	PEDV +, PDCoV +
47	28.1	Fecal Swab	Missouri, Grow-Finish	Not Available	PEDV +, PDCoV -
48	28.6	Oral Fluid	Missouri, Grow-Finish	Not Available	PEDV +, PDCoV +
49	29.8	Fecal Swab	Oklahoma, Grow-Finish	11 Weeks	PEDV +, PDCoV -
50	30	Fecal Swab	Oklahoma, Grow-Finish	12 Weeks	PEDV +, PDCoV -
51	31	Oral Fluid	Missouri, Grow-Finish	Not Available	PEDV +, PDCoV +
52	31.1	Fecal Swab	Iowa, Nursery	13 Weeks	PEDV +, PDCoV -
53	31.7	Fecal Swab	Oklahoma, Grow-Finish	3 Weeks	PEDV +, PDCoV -
54	32	Fecal Swab	Oklahoma, Grow-Finish	18 Weeks	PEDV +, PDCoV -

55	32.9	Oral Fluid	Missouri, Grow-Finish	Not Available	PEDV+
56	33.05	Feces	Bradford, IL/61421	Not Available	1/11/22
57	33.4	Oral Fluid	Missouri, Grow-Finish	Not Available	PEDV+
58	35.1	Feces	Raymond, IL 62560	Not Available	5/9/22
59	35.2	Intestine	Bradford, IL/61421	Not Available	1/11/22
60	35.88	Feces	Bradford, IL/61421	Not Available	1/11/22
61	36.98	Feces	Bradford, IL/61421	Not Available	1/11/22
62	37.21	Feces	Bradford, IL/61421	Not Available	1/11/22
63	37.42	Feces	Not available	Not Available	2025
64	37.42	Fecal Swab#4	North Fork, IL	Not Available	PEDV
65	≥36	Oral Fluid	Indiana	11 Weeks	PEDV-, PDCoV+
66	≥36	Oral Fluid	Iowa, Grow-Finish	6 Months	PEDV-, PDCoV+
67	≥36	Oral Fluid	Illinois	Not Available	PEDV-, PDCoV+
68	≥36	Oral Fluid	Illinois	Not Available	PEDV-, PDCoV+
69	≥36	Oral Fluid	Illinois	Not Available	PEDV-, PDCoV+
70	≥36	Oral Fluid	Illinois	Not Available	PEDV-, PDCoV+
71	≥36	Oral Fluid	Missouri	Not Available	PEDV-, PDCoV+
72	≥36	Oral Fluid	Iowa, Nursery	10 Weeks	PEDV-, PDCoV+
73	≥36	Oral Fluid	Nebraska, Nursery	9 Weeks	PEDV-, PDCoV-
74	≥36	Oral Fluid	Nebraska, Nursery	9 Weeks	PEDV-, PDCoV-
75	≥36	Oral Fluid	Nebraska, Nursery	9 Weeks	PEDV-, PDCoV-
76	≥36	Oral Fluid	Nebraska, Nursery	9 Weeks	PEDV-, PDCoV-
77	≥36	Oral Fluid	Nebraska, Nursery	9 Weeks	PEDV-, PDCoV-
78	34.01	Serum	Not available	Not Available	PRRSV
79	>=36	Fecal Swab	Oklahoma, Sow	2 Years	PEDV-, PDCoV+
80	>=36	Fecal Swab	Oklahoma, Sow	2 Years	PEDV-, PDCoV+
81	>=36	Fecal Swab	Oklahoma, Sow	2 Years	PEDV-, PDCoV+
82	>=36	Fecal Swab	Missouri	Not Available	PEDV-, PDCoV+

83	>=36	Fecal Swab	Missouri	Not Available	PEDV-, PDCoV+
84	>=36	Fecal Swab	Missouri	Not Available	PEDV-, PDCoV+
85	>=36	Fecal Swab	Illinois	Not Available	PEDV-, PDCoV-
86	>=36	Fecal Swab	Illinois	Not Available	PEDV-, PDCoV-
87	>=36	Fecal Swab	Illinois	Not Available	PEDV-, PDCoV-
88	>=36	Fecal Swab	Illinois	Not Available	PEDV-, PDCoV-
89	>=36	Fecal Swab	Illinois	Not Available	PEDV-, PDCoV-
90	23.53	Oral Fluid	Not available	Not Available	SIV
91	33.93	Oral Fluid	Not available	Not Available	SIV
92	32.37	Oral Fluid	Not available	Not Available	PRRSV
93	37.79	Pooled Serum	Not available	Not Available	PRRSV
94	33.29	Oral Fluid	Not available	Not Available	PRRSV
95	30.27	Oral Fluid	Not available	Not Available	PRRSV
96	25.01	Mediastinal Lymph Node	Not available	Not Available	PCV
97	34.96	Pooled Serum	Not available	Not Available	PRRSV
98	38.36	Pooled Serum	Not available	Not Available	PRRSV Suspect
99	PEDV Negative	Fluids squeezed from Swiffer pads used for sampling the surfaces	Not Available	Not Available	Environmental Sample
100	PEDV Negative		Not Available	Not Available	Environmental Sample
101	PEDV Negative		Not Available	Not Available	Environmental Sample
102	PEDV Negative		Not Available	Not Available	Environmental Sample

* SIV (Swine Influenza Virus), PRRSV (Porcine Reproductive and Respiratory Syndrome Virus), and PCV (Porcine Circovirus). qRT-PCR method was used to calculate Ct values.

Table S12. Estimated per test consumable cost and instrumentation requirements for the DNA-Net LFA compared with representative PEDV diagnostic platforms

Platform / method	Main components and readout	Approx consumable cost per test (USD)	Required equipment	Comments on scalability and cost
DNA-Net based PEDV LFA (this work)	DNA-Net with PEDV-N aptamers integrated on LFA setup, AuNP-DNA Net conjugate, visual readout	About 0.8 - 1.5 USD per strip at moderate scale. DNA oligos are bulk ordered at tens to hundreds of nmol which brings the effective cost of all Net strands per strip into the range 0.2 to 0.5 USD. Classical LFA raw materials and AuNPs contribute roughly 0.5 to 1.0 USD, consistent with reported manufacturing costs for lateral flow tests in the cent to low dollar range	None for visual readout, optional low-cost optical reader or smartphone adaptor if semi quantitative analysis is desired	The only additional cost compared with conventional LFA is the multistrand DNA Net, whose per test contribution is small once strands are synthesized in bulk. Assembly is a one pot annealing step that can be batched at milliliter scale and then aliquoted across tens of thousands of strips.
Electrochemical DNA barcode aptasensor[6]	Dual electrode electrochemical chip, electroactive DNA barcode aptamer, signal read by potentiostat	Consumables typically 1.5 to 4 USD per test. Screen printed or microfabricated electrochemical chips are often 1 to 3 USD each at laboratory scale. Reagents and aptamers contribute a further 0.5 to 1 USD. Additional potentiostat about 1,000 to 4,000 USD.	Bench top or portable potentiost at plus laptop or embedded reader. These instruments add several hundred to several thousand USD of capital cost.	Highly sensitive and quantitative but requires electrochemical hardware and trained personnel. For on farm screening the need for an electrochemical reader and single use chips makes the effective per test cost significantly higher than visual LFAs unless very high throughput is achieved at centralized hubs. Best suited for centralized or semi centralized labs with moderate throughput
Latex bead based PEDV LFA[7]	Paper strip, colored latex beads conjugated to anti PEDV mAbs, visual readout	Raw strip materials, antibodies and colored latex conjugates are in the same cost bracket as gold nanoparticle LFAs and may cost around 1 to 3 USD.	None for visual readout	Demonstrates that PEDV LFA with additional filter pads and improved labels can be manufactured and stored at room temperature for at least 56 days. This gives a realistic benchmark for what a DNA-Net based LFA can achieve in terms of stability and cost when produced on similar lines

Commercial PED antigen GICA stripClick or tap here to enter text.	Colloidal gold conjugated monoclonal antibodies on paper strip, visual readout	Vendor price list indicates typical prices around 80 to 120 EUR for boxes of 10 to 20 tests, corresponding to roughly 5 to 10 USD per test at point of purchase.	None for visual readout in the field	These kits set the practical market benchmark for veterinary on farm PEDV testing. Their bill of materials is similar to our DNA Net LFA, but they replace multivalent DNA with protein antibodies and typically achieve higher LODs in viral titer units. Our strip therefore reaches a lower effective viral copy number at a comparable materials cost, with room to reduce cost further if DNA synthesis is scaled.
Gold magnetic nanoparticle SERS immunochromatographic assay[8]	Immunochromatographic strip with gold magnetic nanoparticles, magnetic enrichment, and Raman reporter, Readout by portable or bench top Raman spectrometer	Per test consumable cost estimated in the 2 to 5 USD range, since gold magnetic nanoparticles and Raman tags are more expensive than plain AuNPs. Strip materials are otherwise similar to conventional LFAs. The main economic burden is the capital cost of the Raman spectrometer which can be several thousand USD	Portable or bench top Raman spectrometer plus magnetic stand	Provides excellent analytical sensitivity and multiplexing but is less suited to low resource, high volume on farm screening where instrument free or very low instrument burden formats are preferred. Relative to this, our DNA Net strip preserves the simplicity of visual LFAs while achieving low copy number detection.
qRT-PCR[9]	RNA extraction kit plus qRT PCR reagents, fluorescent readout in real time thermocycler	About 30 USD per test, as per Diagnostic Testing rate listed in Iowa State University[10]	Real-time PCR thermocycler, centrifuge, cold storage, trained staff	Gold standard in terms of analytical sensitivity and genotyping but not suitable for rapid, decentralized screening. Compared with qRT PCR our DNA Net LFA trades one to two orders of magnitude in analytical sensitivity for a much lower per test cost, minimal equipment and a true on farm workflow.

*All cost figures are approximate ranges intended to show the correct order of magnitude. They are based on typical academic or small-scale industrial pricing of oligonucleotides, antibodies, nanoparticles, LFA raw materials, and qPCR reagents, along with publicly visible price lists for commercial kits and general analyses of LFA and molecular test economics[8, 9].

References:

1. Victorious, A.; Zhang, Z.; Chang, D.; Maclachlan, R.; Pandey, R.; Xia, J.; Gu, J.; Hoare, T.; Soleymani, L.; Li, Y., A DNA Barcode-Based Aptasensor Enables Rapid Testing of Porcine Epidemic Diarrhea Viruses in Swine Saliva Using Electrochemical Readout. *Angewandte Chemie International Edition* **2022,** *61* (31).
2. Holstein, C. A.; Griffin, M.; Hong, J.; Sampson, P. D., Statistical Method for Determining and Comparing Limits of Detection of Bioassays. *Analytical Chemistry* **2015,** *87* (19), 9795-9801.
3. Chauhan, N.; Xiong, Y.; Ren, S.; Dwivedy, A.; Magazine, N.; Zhou, L.; Jin, X.; Zhang, T.; Cunningham, B. T.; Yao, S.; Huang, W.; Wang, X., Net-Shaped DNA Nanostructures Designed for Rapid/Sensitive Detection and Potential Inhibition of the SARS-CoV-2 Virus. *Journal of the American Chemical Society* **2023,** *145* (37), 20214-20228.
4. Zou, S.; Wu, L.; Li, G.; Wang, J.; Cao, D.; Xu, T.; Jia, A.; Tang, Y., Development of an accurate lateral flow immunoassay for PEDV detection in swine fecal samples with a filter pad design. *Animal Diseases* **2021,** *1* (1).
5. Zhang, Y.; Xiong, Y.; Xiao, Y., 3dDNA: A Computational Method of Building DNA 3D Structures. *Molecules* **2022,** *27* (18).
6. He, Z.; Duan, X.; Zhao, Z.; Chen, Y.; Fu, C.; Zhang, F.; Wang, J.; Feng, J.; Lin, N.; Chen, H., Rapid on-site diagnosis of PEDV and PoRV co-infection by gold magnetic nanoparticles-based SERS immunochromatography. *Talanta* **2025,** *285*.
7. Wang, L.; Zhang, Y.; Byrum, B., Development and evaluation of a duplex real-time RT-PCR for detection and differentiation of virulent and variant strains of porcine epidemic diarrhea viruses from the United States. *Journal of Virological Methods* **2014,** *207*, 154-157.
8. Wong, R.; Tse, H., *Lateral Flow Immunoassay*. 2009.
9. Alhabbab, R. Y., Economical and Easily Obtainable Tools to Manually Develop Lateral Flow Immunoassay Strips. *ACS Omega* **2023,** *8* (10), 9170-9178.
